# Synthesis and Antimicrobial Characterization of Half-Calycanthaceous Alkaloid Derivatives

**DOI:** 10.3390/molecules21091207

**Published:** 2016-09-09

**Authors:** Shaojun Zheng, Xinping Zhou, Shixun Xu, Rui Zhu, Hongjin Bai, Jiwen Zhang

**Affiliations:** 1 School of Environmental and Chemical Engineering, Jiangsu University of Science and Technology, Zhenjiang 212003, Jiangsu, China; sz281cam@just.edu.cn (S.Z.); zhurui991314@163.com (R.Z.); 2 Key Laboratory of Protection & Utilization of Biological Resources in Tarim Basin of Xinjiang Production and Construction Corps/College of Life Sciences, Tarim University, Alar 843300, Xinjiang, China; zhouzhouxinping@163.com (X.Z.); xsx_89@163.com (S.X.); 3 Key Laboratory of Botanical Pesticide R & D in Shaanxi Province, Yangling 712100, Shaanxi, China

**Keywords:** calycanthaceous alkaloids, synthesis, biological activity, agrochemicals, SAR

## Abstract

A total of 29 novel tetrahydropyrroloindol-based calycanthaceous alkaloid derivatives were synthesized from indole-3-acetonitrile in good yields. The synthesized compounds were evaluated against nine strains of bacteria and a wide range of plant pathogen fungi. Bioassay results revealed that majority of the compounds displayed similar or higher in vitro antimicrobial activities than the positive control. The biological activities also indicated that substituents at R_4_ and R_5_ significantly affect the activities. Notably, compound **c4** was found to be most active among the tested calycanthaceous analogues and might be a novel potential leading compound for further development as an antifungal agent. The results could pave the way for further design and structural modification of calycanthaceous alkaloids as antimicrobial agents.

## 1. Introduction

**Figure 1:**
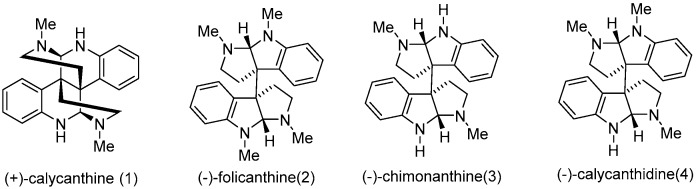
Structures of calycanthaceous alkaloids.

As a part of conventional agriculture, agrochemicals play an important role in the protection of vegetable and cereal crops. However, the unrestricted usage of highly toxic agrochemicals over the past several decades had caused negative effects on environment and poisoned non-targeted species. Therefore, to reduce the negative impacts of agrochemicals, new compounds with high efficacy against target species are desired. Natural-product-based libraries provide a rich source for new agrochemical discovery [1,2,3,4]. Calycanthaceous alkaloids [5,6] (Figure 1), mainly distributed in China, North America and Australia [7,8], are an important class of alkaloids that can be isolated from the roots, leaves, flowers, and fruits of *Chimonanthus praecox* [9], and have demonstrated widespread biological activities such as anticonvulsant, antifungal, antiviral, analgesic, antitumor, and inhibition of melanogenesis properties [10,11,12,13]. Due to their broad spectrum of biological properties, a number of studies towards the synthesis and antimicrobial activity of calycanthaceous alkaloids have been reported [13,14,15,16,17,18,19,20,21,22,23,24,25]. However, the core structure of calycanthaceous alkaloids is underexplored. Therefore, we focused on the structural optimization of tetrahydropyrroloindole. It was hoped that a leading compound could be identified for the discovery of novel antimicrobial agents. Herein, a series of novel calycanthaceous alkaloids analogs were designed and synthesized using indole-3-acetonitrile as the starting material.

To the best of our knowledge, the antimicrobial activities of the synthetic derivatives are reported herein for the first time.

## 2. Results and Discussion

### 2.1. Design and Synthesis of Calycanthaceous Alkaloids Analogues

**Scheme 1:**
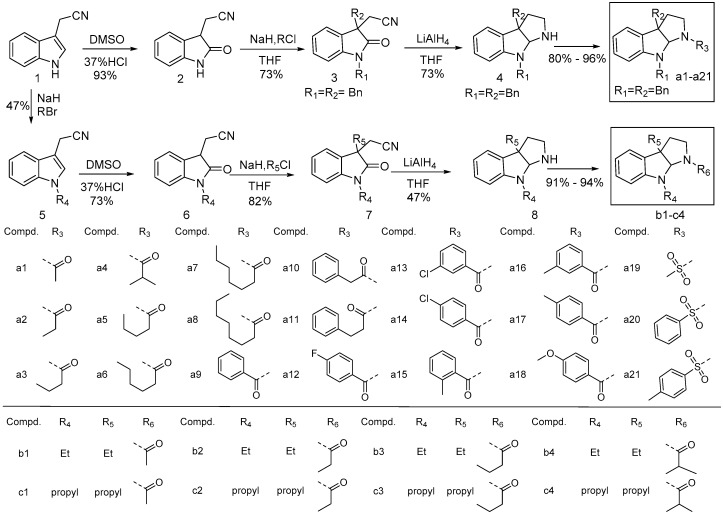
Synthetic route to the title compounds **a1**–**a21** and **b1**–**c4**.

The synthetic route is outlined in Scheme 1. Intermediates **1**–**8** were synthesized according to the corresponding references and their spectral data were consistent with literature values [26,27,28]. The analogs of calycanthaceous alkaloids were synthesized using indole-3-acetonitrile as the starting material via acylation at the N3 position. Twenty nine derivatives were synthesized and characterized by ^1^H-NMR, and ^13^C-NMR spectroscopy and ESI-MS.

### 2.2. Antimicrobial Acitivity

Antibacterial results are shown in Table 1. MIC and MBC values were determined using the microdilution method, with gentamicin and streptomycin as positive controls to evaluate the biological activities of analogues against Gram-positive bacteria (*B. cereus*, *S. aureus*, and *S. epidermidis*).

Table 1 and Table 2 show which of the synthesized compounds exhibited potent in vitro antimicrobial activities against *B. cereus*, *S. aureus*, and *S. epidermidis.* Compounds **a3**, **a7**–**a12**, **a16**, **a20**, **b1**–**b4** and **c1**–**c4** showed higher degrees of activity against *B. cereus* than gentamicin, with **b3** and **a12** being the most effective, with MIC values of 7.81 µg·mL^−1^ and 15.63 µg·mL^−1^, respectively. Compound **a4** showed comparable control efficacy against *S. epidermidis* to that of gentamicin. Noticeably, compound **c4** revealed potent activity against *B. cereus*, *S. aureus*, and *S. epidermidis*, with MIC values of 62.5 µg·mL^−1^, 31.25 µg·mL^−1^, and 31.25 µg·mL^−1^, respectively.

As for inhibitory effects of the synthesized compounds against gram-negative bacteria and gram-positive bacteria, 23 compounds exhibited high degrees of activity against *E. coli*, *S. typhimurium*, and *S. flexneri*. 28 compounds showed high degrees of activity against *E. coli*, *P. aeruginosa*, and *R. solanacearum*. Compounds **a1**, **a8**, **a11**, **a19**, **b1**–**b3**, and **c3**–**c4** exhibited high degrees of activity against more than two kinds of Gram-negative bacteria than that of gentamicin and streptomycin. Noticeably, compound **c4** showed a broad spectrum and remarkably high activity against *E. coli*, *S. typhimurium*, *S. flexneri*, *Escherichia* sp., *P. aeruginosa*, and *R. solanacearum*, with MIC values of 31.25 µg·mL^−1^, 62.5 µg·mL^−1^, 31.25 µg·mL^−1^, 16.5 µg·mL^−1^, 62.5 µg·mL^−1^, and 1.96 µg·mL^−1^, respectively.

Inhibitory effects of calycanthaceous alkaloid analogues against a wide range of plant pathogen fungi are listed in Table 3. MIC and MFC were examined with amphotericin B and carbendazim as a positive controlx, to evaluate the activities of the synthesized calycanthaceous alkaloid analogues against *P. capsici*, *V. dahliae*, *F. oxysperium* sp. *vasinfectum*, *C. orbiculare*, *P. citrinum*, *Cytospora juglandis*, *A. sflavu*, *A. solani*, *C. lunaia*, *F. oxysporum*, and *A. niger*.

It is observed that this series of compounds generally exhibits more effective antimicrobial activity than the positive control. Twenty three compounds exhibited high degrees of activity against *P. capsici*, *V. dahliae*, *F. oxysperium* sp. *vasinfectum*, and *C. orbiculare*. Nineteen compounds showed high degrees of activity against *P. citrinum*, *A. sflavu*, and *A. solani*. Nine of the synthesized compounds manifested high degrees of activity against *C. lunaia*. 27 compounds illustrated high degrees of activity against *F. oxysporum*. Twenty eight compounds manifested high degrees of activity against *A. niger*. Compounds **a11**, **b2**, **b3**, and **c4** manifested higher degrees of activity against *V. dahliae* than amphotericin B and carbendazim, with **c4** being the most effective with a MIC value of 62.5 µg·mL^−1^. Compounds **a3**, **a4**, **a7**–**a8**, **a18**, **b2**–**b3**, and **c1**–**c2** displayed better degrees of activity against *C. lunaia* than that of carbendazim, with **b3** and **a12** being the most effective with MIC values of 62.5 µg·mL^−1^. Compounds **a4**, **a7**, **a8**, **a12**, **a15**, **c1**, **c2**, and **c4** illustrated higher degrees of activity against *F. oxysporum* than that of amphotericin B, with **a4** being the most effective with MIC value of 15.63 µg·mL^−1^.

Compounds **a4**, **a8**, **c1**, and **c2** indicated higher degrees of activity against *C. lunaia* and *F. oxysporum* than that of amphotericin B and carbendazim. Compound **a7** displayed higher degrees of activity against *Cytospora juglandis*, *C. lunaia* and *F. oxysporum* than amphotericin B and carbendazim. Compound **a8** illustrated higher degrees of activity against, *C. lunaia* and *F. oxysporum* than amphotericin B and carbendazim. Compound **b2** manifested higher degrees of activity against *V. dahliae*, *C. lunaia* and *Cytospora juglandis* than amphotericin B and carbendazim. Compound **b3** illustrated higher degrees of activity against *V. dahliae*, and *C .lunaia* than amphotericin B and carbendazim. Especially, compound **c4** displayed higher degrees of activity against *C. orbiculare*, and *C. lunaia* than amphotericin B and carbendazim.

## 3. Materials and Methods

### 3.1. Instruments and Chemicals

All reagents and solvents were reagent grade or purified according to standard methods before use. Analytical thin-layer chromatography (TLC) was performed with silica gel plates using silica gel 60 GF_254_ (Qingdao Haiyang Chemical Co., Ltd., Qingdao, China). Melting points were measured on an Electrothermal digital apparatus (Beijing, China) and were uncorrected. The ^1^H-NMR (500 MHz), and ^13^C−NMR (125 MHz) were obtained on an AM-500 FT-NMR spectrometer (Bruker Corporation, Switzerland) with CDCl_3_ as the solvent and TMS as the internal standard. MS were recorded under ESI conditions using a LCQ Fleet instrument (Thermo Fisher, Waltham, MA, USA). Optical rotation was measured by an Autopol II polarimeter (Rudolph, Hackettstown, NJ, USA). Yields were not optimized. The title compounds were synthesized under a nitrogen atmosphere.

### 3.2. Synthesis

#### 3.2.1. Synthesis of 2-(2-Oxoindolin-3-yl)acetonitrile (**2**)

To a stirred solution of indole-3-acetonitrile (3.12 g, 20 mmol) in DMSO (30 mL) was added hydrogen chloride (37% HCl, V_DMSO_:V_HCl_ = 1:5) dropwise at 0 °C. The resulting mixture was allowed to warm to room temperature. Then the mixture was stirred for 1 h. The solvents were removed to obtain the white solid. The solid was crystallized from acetone to provide the desired product **2** (3.20 g, 93% yield).

#### 3.2.2. Synthesis of 2-(1,3-Dibenzyl-2-oxoindolin-3-yl)acetonitrile (**3**)

To a solution of NaH (1.8 g, 75 mmol) in THF (20 mL) was added the compound **2** (2.58 g, 15 mmol) in THF (10 mL) dropwise at 0 °C. To the resulting mixture was added benzyl chloride (34.5 mmol, 2.3 eq) in THF (5 mL) dropwise at 0 °C. Then the reaction mixture was quenched with ammonium chloride (1 mL), and extracted three times with ethyl acetate. The organic extracts were combined, washed with brine, dried over Na_2_SO_4_, and concentrated. Purification by flash chromatography on silica (petroleum ether–ethyl acetate = 4:1) gel afforded compound **3** (3.84 g, 73% yield).

#### 3.2.3. Synthesis of 3a,8-Dibenzyl-1,2,3,3a,8,8a-hexahydropyrrolo[2,3-*b*]indole (**4**)

To compound **3** (3.52 g, 10 mmol) in THF (20 mL) was added LiAlH_4_ (60 mmol, 7.5 eq) at 0 °C. The resulting mixture was refluxed for 2 h, then, it was allowed to reach room temperature and stirred for another 1 h, quenched with H_2_O (1 mL), and extracted three times with ethyl acetate. The organic extracts were combined, washed with brine, dried over Na_2_SO_4_, and concentrated. Purification by flash chromatography (petroleum ether–ethyl acetate = 2:1) on silica gel afforded compound **4** (2.17 g, 63% yield).

#### 3.2.4. Synthesis of Compounds **a1**–**a21**

To compound **4** in pyridine (10 mL) was added the corresponding desired reagent at 0 °C. The resulting mixture was refluxed for 2 h. The resulting mixture was allowed to warm to room temperature and then stirred for a 1 h. Then, the reaction mixture was quenched with methanol (1 mL), and extracted three times with ethyl acetate. The organic extracts were combined, washed with brine, dried over Na_2_SO_4_, and concentrated. Purification by flash chromatography on silica gel afforded the compounds **a1**–**a21** in yields from 80% to 96%. (Characterization data see Supplementary Materials)

#### 3.2.5. Synthesis of 2-(1-Ethyl-1*H*-indol-3-yl)acetonitrile (**5**)

To a solution of NaH (1.8 g, 75 mmol) in THF (20 mL) was added indole-3-acetonitrile (3.12 g, 20 mmol) in DMSO (30 mL) dropwise at 0 °C. The resulting mixture was allowed to reach room temperature for a further 30 min. Then, the resulting mixture was added the compound RBr in THF (5 mL) dropwise at 0 °C. The reaction mixture was quenched with ammonium chloride (1 mL), and extracted three times with ethyl acetate. The organic extracts were combined, washed with brine, dried over Na_2_SO_4_, and concentrated. Purification by flash chromatography (petroleum ether–ethyl acetate = 4:1) on silica gel afforded the compound **5** (1.47 g, 47% yield).

#### 3.2.6. Synthesis of 2-(1-Ethyl-2-oxoindolin-3-yl)acetonitrile (**6**)

To a stirred solution of **5** (3.12 g, 20 mmol) in DMSO (30 mL) was added hydrogen chloride (37% HCl, V_DMSO_:V_HCl_ = 1:5) dropwise at 0 °C. The resulting mixture was allowed to warm to room temperature and then stirred for 1 h. Then, the solvent was removed. Purification by flash chromatography (petroleum ether–ethyl acetate = 2.5:1) on silica gel afforded the compound **6** (1.17 g, 73% yield).

#### 3.2.7. Synthesis of 2-(1,3-Diethyl-2-oxoindolin-3-yl)acetonitrile (**7**)

To a solution of NaH (1.8 g, 75 mmol) in THF (20 mL) was added compound **6** (2.58 g, 15 mmol) in THF (10 mL) dropwise at 0 °C. The resulting mixture was added the compound RX in THF (5 mL) dropwise at 0 °C. The reaction mixture was quenched with ammonium chloride (1 mL), and extracted three times with ethyl acetate. The organic extracts were combined, washed with brine, dried over Na_2_SO_4_, and concentrated. Purification by flash chromatography (petroleum ether–ethyl acetate = 3:1) on silica gel afforded the compound **7** (1.09 g, 82% yield).

#### 3.2.8. Synthesis of 3a,8-Diethyl-1,2,3,3a,8,8a-hexahydropyrrolo[2,3-*b*]indole (**8**)

To compound **7** (0.8 g, 2.8 mmol) in THF (20 mL) was added LiAlH_4_ (21 mmol,7.5 eq) at 0 °C. The resulting mixture was refluxed for 2 h, then allowed to warm to room temperature for a further 1 h. Then, the reaction mixture was quenched with H_2_O (1 mL), and extracted three times with ethyl acetate. The organic extracts were combined, washed with brine, dried over Na_2_SO_4_, and concentrated. Purification by flash chromatography (petroleum ether–ethyl acetate = 1.5:1) on silica gel afforded compound **8** (0.45 g, 47% yield).

#### 3.2.9. Synthesis of Compounds **b1**–**c4**

To a stirred solution of compound **8** in pyridine (10 mL) was added the corresponding desired reagents at 0 °C. The resulting mixture was heated to reflux for 2 h. The resulting mixture was allowed to warm to rt. Then, the mixture was stirred for 1 h. The reaction mixture was quenched with methanol (1 mL), and extracted three times with ethyl acetate. The organic extracts were combined, washed with brine, dried over Na_2_SO_4_, and concentrated. Purification by flash chromatography on silica gel afforded the compounds **b1**–**c4** in yields, from 91% to 94%). (Characterization data see Supplementary Materials)

### 3.3. Biological Activity

The antimicrobial activity of calycanthaceous alkaloids analogues were measured according to the previously reported method [29,30].

The tested compounds dissolved in 5% dimethyl sulfoxide (DMSO), to a concentration of 1.02 mg/mL, 100 μL of the solutions were added to the first well and serially diluted from first well by taking 100 μL into second. This two-fold dilution was continued down the plate and 100 μL from the 8th column of the plated discarded. The 9th column of the plate was reserved for negative control wells (without inocula) and the 10th column, for the positive growth control wells (without antibacterial agent). The antibacterial concentrations were 256, 128, 64, 32, 16, 8, 4 and 2 μg/mL, respectively. The antibacterial test plates were incubated aerobically at 37 °C for 24 h, the antifungal test plates were incubated aerobically at 28 °C for 48 h. The MICs, MBC and MFC were examined. MBC and MFC were determined by plating 10 μL from each negative well and from the positive growth control on LB Agar and Sabouraud Dextrose Agar. MBC and MFC were defined as the lowest concentration yielding negative subcultures or only on colony. All tests were performed in triplicate and repeated if the results differed.

## 4. Conclusions

Twenty nine novel tetrahydropyrroloindole-based calycanthaceous alkaloid derivatives were synthesized using indole-3-acetonitrile as the starting material via acylation at the N3 position, and their antimicrobial activity against nine strains of bacteria and a wide range of plant pathogen fungi were evaluated. According to the bioassay results, most of the title compounds showed moderate to excellent activities against the selected bacteria and a wide variety of plant pathogen fungi and were more effective than the positive controls. The compounds with substituted aliphatic groups at positions R4 and R5, showed higher antimicrobial activities than with the aromatic substituents. This suggested that substituents at R_4_ and R_5_ significantly affect the activities of title calycanthaceous alkaloid analogues. Notably, compound **c4** displayed the broadest and most effective activities among the tested calycanthaceous analogues and might be a novel potential leading compound for further development of antifungal agents. These results will pave the way for further design, structural modification, and development of calycanthaceous alkaloids as antimicrobial agents.

## Figures and Tables

**Table 1:** Antibacterial activity of calycanthaceous alkaloids derivatives against *B. cereus*, *S. aureus*, and *S. epidermidis*.

Compounds	MIC μg/mL			MBC μg/mL		
	*B. cereus*	*S. aureus*	*S. epidermidis*	*B. cereus*	*S. aureus*	*S. epidermidis*
**a1**	−	256	62.5	−	−	125
**a2**	−	256	62.5	−	−	125
**a3**	125	−	−	256	−	−
**a4**	−	−	62.5	−	−	125
**a5**	−	−	−	−	−	−
**a6**	256	−	−	−	−	−
**a7**	31.25	256	62.5	62.5	−	125
**a8**	62.5	256	125	125	−	256
**a9**	125	−	−	256	−	−
**a10**	125	−	−	256	−	−
**a11**	31.25	125	62.5	62.5	256	125
**a12**	15.63	−	−	31.25	−	−
**a13**	−	−	−	−	−	−
**a14**	256	−	−	−	−	−
**a15**	256	−	−	−	−	−
**a16**	62.5	−	−	125	−	−
**a17**	−	−	−	−	−	−
**a18**	−	−	−	−	−	−
**a19**	−	256	62.5	−	−	125
**a20**	125	256	125	256	−	256
**a21**	−	−	−	−	−	−
**b1**	62.5	125	125	125	256	256
**b2**	31.25	125	125	62.5	256	256
**b3**	7.81	125	125	15.63	256	256
**b4**	31.25	256	125	62.5	−	256
**c1**	62.5	125	62.5	125	256	125
**c2**	125	125	62.5	256	256	125
**c3**	62.5	125	125	125	256	256
**c4**	62.5	31.25	31.25	125	62.5	62.5
**gentamicin**	−	62.5	15.63	−	125	31.25
**streptomycin**	1.96	15.63	31.25	3.9	31.25	62.5

Note: The gentamicin and streptomycin were used as the positive controls; “−” means no inhibition effect. MIC: Minimal Inhibitory Concentration; MBC: Minimum Bactericidal Concentration.

**Table 2:** Antibacterial activity of calycanthaceous alkaloids derivatives against *E. coli*, *S. typhimurium*, *S. flexneri*, *Escherichia* sp., *P. aeruginosa*, and *R. solanacearum*.

Compounds	MIC μg/mL						MBC μg/mL					
	*E.c.*	*S.t.*	*S.f.*	*E.s.*	*P.a.*	*R.s.*	*E.c.*	*S.t.*	*S.f.*	*E.s.*	*P.a.*	*R.s.*
**a1**	125	31.25	62.5	62.5	256	62.5	256	62.5	125	125	−	125
**a2**	125	125	125	125	125	−	256	256	256	256	250	−
**a3**	−	−	−	−	125	125	−	−	−	−	250	256
**a4**	−	125	125	−	125	7.81	−	256	256	−	250	15.63
**a5**	−	−	−	−	125	125	−	−	−	−	250	256
**a6**	−	−	−	−	62.5	256	−	−	−	−	125	−
**a7**	62.5	125	−	125	62.5	7.81	125	256	−	256	125	15.63
**a8**	62.5	125	125	125	62.5	31.25	125	256	256	256	125	62.5
**a9**	−	−	−	−	125	15.63	−	−	−	−	250	31.25
**a10**	−	−	−	−	62.5	125	−	−	−	−	125	256
**a11**	62.5	125	62.5	125	62.5	15.63	125	256	125	256	125	31.25
**a12**	−	125	125	−	125	7.81	−	256	256	−	250	15.63
**a13**	−	125	125	−	−	−	−	256	256	−	−	−
**a14**	−	125	125	−	62.5	256	−	256	256	−	125	−
**a15**	−	125	125	−	125	256	−	256	256	−	250	−
**a16**	−	125	256	−	62.5	125	−	256	−	−	125	256
**a17**	−	62.5	62.5	−	62.5	62.5	−	125	125	−	125	125
**a18**	−	−	−	−	7.81	125	−	−	−	−	15.63	256
**a19**	62.5	125	62.5	125	15.63	256	125	256	125	256	31.25	−
**a20**	62.5	125	−	125	31.25	62.5	125	256	−	256	62.5	125
**a21**	−	125	62.5	−	125	−	−	256	125	−	250	−
**b1**	62.5	125	62.5	−	125	256	125	256	125	−	250	−
**b2**	62.5	125	125	125	125	−	125	256	256	256	250	−
**b3**	62.5	125	62.5	62.5	62.5	125	125	256	125	125	125	265
**b4**	62.5	−	−	62.5	7.81	125	125	−	−	125	15.63	256
**c1**	125	125	−	125	31.25	125	256	256	−	256	62.5	256
**c2**	125	125	−	125	15.63	125	256	256	−	256	31.25	256
**c3**	125	62.5	−	62.5	15.63	256	256	125	−	125	31.25	−
**c4**	31.25	62.5	31.25	16.5	62.5	1.96	62.5	125	62.5	31.25	125	3.9
**gentamicin**	125	125	256	256	−	−	256	256	−	−	−	−
**streptomycin**	125	125	62.5	7.81	1.96	3.9	256	256	125	15.63	3.9	7.81

Note: The gentamicin and streptomycin were used as the positive control. “−” means no inhibition effect. *E.c.: E. coli*; *S.t.: S. typhimurium*; *S.f.: S. flexneri*; *E.S.: Escherichia* sp.; *P.a.: P. aeruginosa* and *R.s.: R. solanacearum*. MIC: Minimal Inhibitory Concentration; MBC: Minimum Bactericidal Concentration.

**Table 3:** Inhibitory effect of synthesized compounds against a wide variety of plant pathogen fungi.

Compounds	MIC(µg/mL)											MFC(µg/mL)										
	*P.ca.*	*V.d.*	*F.s.*	*C.o.*	*P.c.*	*C.j*.	*A.sf.*	*A.s.*	*C.l.*	*F.o.*	*A.n.*	*P.ca.*	*V.d.*	*F.s.*	*C.o.*	*P.c.*	*C.j.*	*A.sf.*	*A.s.*	*C.l.*	*F.o.*	*A.n.*
**a1**	31.25	−	256	125	−	125	256	256	−	−	62.5	62.5	−	−	256	−	256	−	−	−	−	125
**a2**	256	−	256	125	−	62.5	256	−	−	256	−	−	−	−	256	−	125	−	−	−	−	−
**a3**	256	−	−	−	−	−	256	256	62.5	125	256	−	−	−	−	−	−	−	−	125	256	−
**a4**	−	−	−	−	−	−	256	256	62.5	15.63	256	−	−	−	−	−	−	−	−	125	31.25	−
**a5**	−	−	256	−	−	−	−	−	−	−	256	−	−	−	−	−	−	−	−	−	−	−
**a6**	−	−	256	−	−	−	−	−	−	125	125	−	−	−	−	−	−	−	−	−	256	256
**a7**	−	256	256	−	−	125	−	256	125	31.25	256	−	−	−	−	−	256	−	−	256	62.5	−
**a8**	−	256	−	−	−	256	−	125	125	62.5	125	−	−	−	−	−	−	−	256	256	125	256
**a9**	−	−	−	−	−	−	−	256	−	256	256	−	−	−	−	−	−	−	−	−	−	−
**a10**	−	−	−	−	−	125	−	−	−	125	256	−	−	−	−	−	256	−	−	−	256	−
**a11**	−	125	−	−	−	256	−	−	−	125	256	−	256	−	−	−	−	−	−	−	256	−
**a12**	−	−	−	256	−	−	−	256	−	62.5	125	−	−	−	−	−	−	−	−	−	125	256
**a13**	−	−	−	−	−	−	−	−	−	256	125	−	−	−	−	−	−	−	−	−	−	256
**a14**	−	−	−	−	−	−	−	−	−	125	256	−	−	−	−	−	−	−	−	−	256	−
**a15**	−	−	256	−	−	−	−	−	−	62.5	256	−	−	−	−	−	−	−	−	−	125	−
**a16**	−	−	256	−	−	256	−	−	−	125	125	−	−	−	−	−	−	−	−	−	256	256
**a17**	256	−	125	256	−	−	−	−	−	256	125	−	−	256	−	−	−	−	−	−	−	256
**a18**	256	−	256	256	−	256	−	−	125	125	62.5	−	−	−	−	−	−	−	−	256	256	125
**a19**	256	−	256	256	−	−	−	−	−	125	256	−	−	−	−	−	−	−	−	−	256	−
**a20**	−	−	125	256	−	−	−	−	−	125	62.5	−	−	256	−	−	−	−	−	−	256	125
**a21**	−	−	−	−	−	−	−	−	−	125	125	−	−	−	−	−	−	−	−	−	256	256
**b1**	125	−	256	256	125	−	256	−	−	125	125	256	−	−	−	256	−	−	−	−	256	256
**b2**	62.5	125	256	125	125	125	256	125	125	256	62.5	125	256	−	256	256	256	−	256	256	−	125
**b3**	125	125	−	256	125	−	256	−	125	125	62.5	256	256	−	−	256	−	−	−	256	256	125
**b4**	125	−	256	256	−	−	−	−	−	125	62.5	256	−	−	−	−	−	−	−	−	256	125
**c1**	62.5	−	−	125	125	256	−	−	125	62.5	125	125	−	−	256	256	−	−	−		125	256
**c2**	31.25	−	−	125	125	−	62.5	256	125	31.25	31.25	62.5	−	−	256	256	−	125	−	256	62.5	62.5
**c3**	256	−	256	256	125	−	125	−	−	256	125	−	−	−	−	256	−	256	−	−	−	256
**c4**	31.25	62.5	256	62.5	62.5	−	15.63	−	−	31.25	125	62.5	125	−	125	125	−	31.25	−	−	62.5	256
**A.**	3.9	62.5	256	125	15.63	256	7.81	−	7.81	125	0.97	7.81	125	−	256	31.25	−	15.63	−	15.63	256	1.93
**C.**	1.95	256	125	0.97	0.97	62.5	0.97	−	−	7.81	−	3.9	−	256	1.95	1.95	125	1.95	−	−	15.63	−

Notes: “−” means no inhibition effect. Amphotericin B and Carbendazim were used as the positive control. *P.ca.*: *P. capsici*, *V.d.*: *V. dahlia*, *F.s.*: *F. oxysperium* sp. *vasinfectum*, *C.o.*: *C. orbiculare*, *P.c.*: *P. citrinum*, *C.j.*: *Cytospora juglandis*, *A.sf.*: *A. sflavu*, *A.s.*: *A. solani*, *C.l.*: *C. lunaia*, *F.o.*: *F. oxysporum*, *A.n.*: *A. niger*, *A.*: *Amphotericin B*, *C.*: *Carbendazim*. MIC: minimal inhibitory concentration; MBC: Minimum Bactericidal Concentration.

## Supplementary Materials

Supplementary materials can be accessed at: http://www.mdpi.com/1420-3049/21/9/1207/s1.
