# Supplementary material for: Synthesis and Antimicrobial Characterization of Half-Calycanthaceous Alkaloid Derivatives
Source: Molecules. 2016 Sep 9;21(9):1207. doi: 10.3390/molecules21091207 (PMC6273825; doi:10.3390/molecules21091207)
Supplement: Supplementary file 1 [file molecules-21-01207-s001.pdf]

Supplementary Materials: Synthesis and Antimicrobial Characterization of Half-Calycanthaceous Alkaloid Derivatives

Shaojun Zheng, Xinping Zhou, Shixun Xu, Rui Zhu, Hongjin Bai and Jiwen Zhang

1. Spectroscopic Data

1-(3a,8-Dibenzyl-3,3a,8,8a-tetrahydropyrrolo[2,3-b]indol-1(2H)-yl)ethan-1-one (**a1**): Reagents: acetic anhydride. Orange oily liquid, $[\alpha]_D^{25}$ = −10.228, petroleum ether:ethyl acetate = 3:1, 96% Yield, ^{1}H-NMR ($CDCl_3$, 500 MHz), δ 7.21–7.16 (m, 6H), 7.06–7.04 (m, 2H), 7.02–6.99 (m, 1H), 6.93–6.89 (m, 3H), 6.67–6.64 (m, 1H), 6.18 (d, 1H, *J* = 10.00 Hz), 5.77 (s, 1H), 4.64, 4.52 (Abq, 2H, *J* = 15.00 Hz), 3.47–3.43 (m, 1H), 3.28–3.21 (m, 1H), 3.04, 2.87 (Abq, 2H, *J*=15.00 Hz), 2.21–2.17 (m, 2H), 1.92 (s, 3H). ^{13}C-NMR ($CDCl_3$, 125 MHz), δ 169.85 (C), 150.44 (C), 139.32 (C), 137.07 (C), 131.70 (C), 130.12 (CH × 2), 128.56 (CH), 128.25 (CH × 2), 128.08 (CH × 2), 126.93 (CH × 2), 126.58 (CH), 126.51 (CH), 123.11 (CH), 117.11 (CH), 106.52 (CH), 82.62 (CH), 56.51 (C), 49.96 (CH_2), 47.16 (CH_2), 44.47 (CH_2), 37.31 (CH_2), 22.71 (CH_3). ESI-MS: $[M + Na]^+$ Found 382.20.

1-(3a,8-Dibenzyl-3,3a,8,8a-tetrahydropyrrolo[2,3-b]indol-1(2H)-yl)propan-1-one (**a2**): Reagents: propionic anhydride. Orange oily liquid, $[\alpha]_D^{25}$ = −13.530, petroleum ether:ethyl acetate = 4:1, 91% Yield, ^{1}H-NMR ($CDCl_3$, 500 MHz), δ 7.21–7.16 (m, 6H), 7.08–7.04 (m, 2H), 7.02–6.99 (m, 1H), 6.93–6.91 (m, 2H), 6.88–6.87 (m, 1H), 6.66–6.63 (m, 1H), 6.20 (d, 1H, *J* = 5.00 Hz), 5.79 (s, 1H), 4.61, 4.54 (Abq, 2H, *J* = 20.00 Hz), 3.50–3.46 (m, 1H), 3.28–3.17 (m, 1H), 3.03, 2.85 (Abq, 2H, *J* = 15.00 Hz), 2.20–2.04 (m, 4H), 1.03 (s, 3H). ^{13}C-NMR ($CDCl_3$, 125 MHz), δ 173.02 (C), 150.51 (C), 139.46 (C), 137.12 (C), 131.78 (C), 130.14 (CH × 2), 128.54 (CH), 128.21 (CH × 2), 128.04 (CH × 2), 127.00 (CH × 2), 126.55 (CH), 126.50 (CH), 123.18 (CH), 117.04 (CH), 106.51 (CH), 82.91 (CH), 56.29 (C), 50.12 (CH_2), 46.18 (CH_2), 44.55 (CH_2), 37.34 (CH_2), 27.98 (CH_2), 8.94 (CH_3). ESI-MS: $[M + Na]^+$ Found 396.22.

1-(3a,8-Dibenzyl-3,3a,8,8a-tetrahydropyrrolo[2,3-b]indol-1(2H)-yl)butan-1-one (**a3**): Reagents: butyric anhydride. Yellow oily liquid, $[\alpha]_D^{25}$ = 14.129, petroleum ether:ethyl acetate = 3:1, 86% Yield, ^{1}H-NMR ($CDCl_3$, 500 MHz), δ 7.21–7.14 (m, 6H), 7.07–7.05 (m, 2H), 7.01–6.98 (m, 1H), 6.93–6.91 (m, 2H), 6.89–6.87 (m, 1H), 6.66–6.63 (m, 1H), 6.17 (d, 1H, *J* = 10.00 Hz), 5.81 (s, 1H), 4.59, 4.53 (Abq, 2H, *J* = 15.00 Hz), 3.53–3.49 (m, 1H), 3.25–3.18 (m, 1H), 3.05, 2.86 (Abq, 2H, *J* = 15.00 Hz), 2.21–2.06 (m, 4H), 1.59–1.51 (m, 2H), 0.88 (s, 3H). ^{13}C-NMR ($CDCl_3$, 125 MHz), δ 172.33 (C), 150.56 (C), 139.39 (C), 137.13 (C), 131.75 (C), 130.12 (CH × 2), 128.53 (CH), 128.21 (CH × 2), 128.04 (CH × 2), 126.94 (CH × 2), 126.55 (CH), 126.47 (CH), 123.19 (CH), 117.06 (CH), 106.49 (CH), 82.87 (CH), 56.32 (C), 50.14 (CH_2), 46.35 (CH_2), 44.62 (CH_2), 37.48 (CH_2), 36.77 (CH_2), 18.20 (CH_2), 13.88 (CH_3). ESI-MS: $[M + Na]^+$ Found 410.24.

1-(3a,8-Dibenzyl-3,3a,8,8a-tetrahydropyrrolo[2,3-b]indol-1(2H)-yl)-2-methylpropan-1-one (**a4**): Reagents: isobutyric anhydride. Orange oily liquid, $[\alpha]_D^{25}$ = −12.220, mp 118–120 °C, petroleum ether:ethyl acetate = 3:1, 92% Yield, ^{1}H-NMR ($CDCl_3$, 500 MHz), δ 7.20–7.14 (m, 6H), 7.06–7.05 (m, 2H), 7.01–6.98 (m, 1H), 6.94–6.92 (m, 2H), 6.89–6.87 (m, 1H), 6.66–6.63 (m, 1H), 6.15 (d, 1H, *J* = 5.00 Hz), 5.84 (s, 1H), 4.55, 4.48 (Abq, 2H, *J* =15.00 Hz), 3.63–3.59 (m, 1H), 3.28–3.22 (m, 1H), 3.07, 2.87 (Abq, 2H, *J* = 15.00 Hz), 2.55–2.49 (m, 1H), 2.25–2.15 (m, 2H), 1.03, 0.95 (dd, 6H, *J* = 5.00 Hz). ^{13}C-NMR ($CDCl_3$, 125 MHz), δ 176.14 (C), 150.75 (C), 139.50 (C), 137.18 (C), 131.69 (C), 130.08 (CH × 2), 128.52 (CH), 128.17 (CH × 2), 128.00 (CH × 2), 126.88 (CH × 2), 126.54 (CH), 126.43 (CH), 123.25 (CH), 117.01 (CH), 106.39 (CH), 83.18 (CH), 56.27 (C), 50.32 (CH_2), 46.04 (CH_2), 44.86 (CH_2), 37.84 (CH_2), 32.18 (CH), 19.11 (CH_3), 18.64 (CH_3). ESI-MS: $[M + Na]^+$ Found 410.24.

1-(3a,8-Dibenzyl-3,3a,8,8a-tetrahydropyrrolo[2,3-b]indol-1(2H)-yl)pentan-1-one (**a5**): Reagents: valeric acid. Colorless oil, $[\alpha]_D^{25}$ = −16.539, petroleum ether:ethyl acetate = 4:1, 87% Yield, ^{1}H-NMR ($CDCl_3$, 500 MHz), δ 7.21–7.15 (m, 6H), 7.07–7.06 (m, 2H), 7.01–6.98 (m, 1H), 6.93–6.91 (m, 2H), 6.89–6.87 (m, 1H), 6.66–6.63 (m, 1H), 6.18 (d, 1H, *J*= 5.00 Hz), 5.81 (s, 1H), 4.58, 4.53 (Abq, 2H, *J* =15.00 Hz), 3.52–3.48 (m, 1H), 3.24–3.18 (m, 1H), 3.05, 2.86 (Abq, 2H, *J* = 15.00 Hz), 2.23–2.06 (m, 4H), 1.52–1.46 (m, 2H), 1.23–1.30 (m,

2H), 0.88 (s, 3H). ^{13}C-NMR ($CDCl_3$, 125 MHz), δ 172.47 (C), 150.58 (C), 139.43 (C), 137.14 (C), 131.76 (C), 130.13 (CH × 2), 128.53 (CH), 128.21 (CH × 2), 128.04 (CH × 2), 126.94 (CH × 2), 126.55 (CH), 126.47 (CH), 123.18 (CH), 117.03 (CH), 106.46 (CH), 82.90 (CH), 56.32 (C), 50.14 (CH_2), 46.36 (CH_2), 44.62 (CH_2), 37.49 (CH_2), 34.60 (CH_2), 26.92 (CH_2), 22.46 (CH_2), 13.88 (CH_3). ESI-MS: $[M + Na]^+$ Found 424.25.

1-(3a,8-Dibenzyl-3,3a,8,8a-tetrahydropyrrolo[2,3-b]indol-1(2H)-yl)hexan-1-one (**a6**): Reagents: caproic acid. Yellow oily liquid, $[\alpha]_D^{25}$ = −14.338, petroleum ether:ethyl acetate = 3:1, 86% Yield, ^{1}H-NMR ($CDCl_3$, 500 MHz), δ 7.19–7.15 (m, 6H), 7.07–7.06 (m, 2H), 7.01–6.98 (m, 1H), 6.93–6.92 (m, 2H), 6.89–6.87 (m, 1H), 6.66–6.63 (m, 1H), 6.18 (d, 1H, *J* =5.00 Hz), 5.81 (s, 1H), 4.58, 4.53 (Abq, 2H, *J* =15.00 Hz), 3.52–3.48 (m, 1H), 3.24–3.18 (m, 1H), 3.05, 2.86 (Abq, 2H, *J* = 15.00 Hz), 2.19–2.05 (m, 4H), 1.54–1.48 (m, 2H), 1.30–1.22 (m, 4H), 0.86 (s, 3H). ^{13}C-NMR ($CDCl_3$, 125 MHz), δ 172.49 (C), 150.60 (C), 139.46 (C), 137.16 (C), 131.78 (C), 130.13 (CH × 2), 128.54 (CH), 128.21 (CH × 2), 128.05 (CH × 2), 126.95 (CH × 2), 126.56 (CH), 126.48 (CH), 123.18 (CH), 117.04 (CH), 106.48 (CH), 82.94 (CH), 56.32 (C), 50.18 (CH_2), 46.35 (CH_2), 44.63 (CH_2), 37.52 (CH_2), 34.87 (CH_2), 31.56 (CH_2), 24.50 (CH_2), 22.45 (CH_2), 13.95 (CH_3). ESI-MS: $[M + Na]^+$ Found 438.27.

1-(3a,8-Dibenzyl-3,3a,8,8a-tetrahydropyrrolo[2,3-b]indol-1(2H)-yl)heptan-1-one (**a7**): Reagents: heptanoic acid. Orange oily liquid, $[\alpha]_D^{25}$ = −10.441, petroleum ether:ethyl acetate = 3:1, 87% Yield, ^{1}H-NMR ($CDCl_3$, 500 MHz), δ 7.19–7.15 (m, 6H), 7.07–7.05 (m, 2H), 7.01–6.98 (m, 1H), 6.93–6.92 (m, 2H), 6.89–6.87 (m, 1H), 6.65–6.63 (m, 1H), 6.17 (d, 1H, *J* = 10.00 Hz), 5.81 (s, 1H), 4.58, 4.53 (Abq, 2H, *J* =15.00 Hz), 3.52–3.48 (m, 1H), 3.24–3.18 (m, 1H), 3.05, 2.86 (Abq, 2H, *J* = 15.00 Hz), 2.20–2.06 (m, 4H), 1.51–1.47 (m, 2H), 1.26–1.24 (m, 6H), 0.86 (s, 3H). ^{13}C-NMR ($CDCl_3$, 125 MHz), δ 172.56 (C), 150.60 (C), 139.44 (C), 137.15 (C), 131.76 (C), 130.13 (CH × 2), 128.54 (CH), 128.21 (CH × 2), 128.05 (CH × 2), 126.95 (CH × 2), 126.56 (CH), 126.48 (CH), 123.18 (CH), 117.04 (CH), 106.48 (CH), 82.93 (CH), 56.33 (C), 50.16 (CH_2), 46.37 (CH_2), 44.63 (CH_2), 37.52 (CH_2), 34.92 (CH_2), 31.59 (CH_2), 29.04 (CH_2), 24.78 (CH_2), 22.50 (CH_2), 14.04 (CH_3). ESI-MS: $[M + Na]^+$ Found 452.28.

1-(3a,8-Dibenzyl-3,3a,8,8a-tetrahydropyrrolo[2,3-b]indol-1(2H)-yl)octan-1-one (**a8**): Reagents: octanoic acid. Yellow oily liquid, $[\alpha]_D^{25}$ = −15.900, petroleum ether:ethyl acetate = 3:1, 85% Yield, ^{1}H-NMR ($CDCl_3$, 500 MHz), δ 7.19–7.15 (m, 6H), 7.07–7.05 (m, 2H), 7.01–6.98 (m, 1H), 6.93–6.92 (m, 2H), 6.89–6.87 (m, 1H), 6.65–6.62 (m, 1H), 6.17 (d, 1H, *J* = 10.00 Hz), 5.81 (s, 1H), 4.58, 4.53 (Abq, 2H, *J* =15.00 Hz), 3.52–3.48 (m, 1H), 3.24–3.18 (m, 1H), 3.05, 2.86 (Abq, 2H, *J* = 15.00 Hz), 2.20–2.04 (m, 4H), 1.51–1.48 (m, 2H), 1.26–1.24 (m, 8H), 0.87 (s, 3H). ^{13}C-NMR ($CDCl_3$, 125 MHz), δ 172.56 (C), 150.59 (C), 139.44 (C), 137.15 (C), 131.76 (C), 130.13 (CH × 2), 128.54 (CH), 128.21 (CH × 2), 128.05 (CH × 2), 126.95 (CH × 2), 126.56 (CH), 126.48 (CH), 123.18 (CH), 117.04 (CH), 106.48 (CH), 82.92 (CH), 56.33 (C), 50.15 (CH_2), 46.38 (CH_2), 44.63 (CH_2), 37.51 (CH_2), 34.92 (CH_2), 29.33 (CH_2), 28.92 (CH_2), 24.82 (CH_2), 22.62 (CH_2), 14.08 (CH_3). ESI-MS: $[M+Na]^+$ Found 466.30.

(3a,8-Dibenzyl-3,3a,8,8a-tetrahydropyrrolo[2,3-b]indol-1(2H)-yl)(phenyl)methanone (**a9**): Reagents: benzoic acid. Yellow oily liquid, $[\alpha]_D^{25}$ = −10.441, mp 152–154 °C, petroleum ether:ethyl acetate = 3:1, 81% Yield, ^{1}H-NMR ($CDCl_3$, 500 MHz), δ 7.40–7.38 (m, 3H), 7.35–7.32 (m, 2H), 7.23–7.16 (m, 6H), 7.05–7.02 (m, 3H), 6.96–6.94 (m, 2H), 6.89–6.88 (m, 1H), 6.69–6.66 (m, 1H), 6.20 (d, 1H, *J* = 5.00 Hz), 6.08 (s, 1H), 4.68, 4.49 (Abq, 2H, *J* = 15.00 Hz), 3.55–3.51 (m, 1H), 3.38–3.32 (m, 1H), 3.14, 2.93 (Abq, 2H, *J* = 10.00 Hz), 2.19–2.08 (m, 2H). ^{13}C-NMR ($CDCl_3$, 125 MHz), δ 170.49 (C), 150.83 (C), 139.19 (C), 137.18 (C), 136.09 (C), 131.24 (C), 130.29 (CH), 130.12 (CH × 2), 128.63 (CH), 128.30 (CH × 2), 128.15 (CH × 2), 128.05 (CH × 2), 127.50 (CH × 2), 126.80 (CH × 2), 126.56 (CH), 126.53 (CH), 123.47 (CH), 117.11 (CH), 106.29 (CH), 82.63 (CH), 56.64 (C), 49.48 (CH_2), 44.96 (CH_2), 37.97 (CH_2), 25.61 (CH_2). ESI-MS: $[M + Na]^+$ Found 444.22.

1-(3a,8-Dibenzyl-3,3a,8,8a-tetrahydropyrrolo[2,3-b]indol-1(2H)-yl)-2-phenylethan-1-one (**a10**): Reagents: phenyl acetic acid. Yellow oily liquid, petroleum ether:ethyl acetate = 5:1, 80% Yield, $[\alpha]_D^{25}$ = −3.093, ^{1}H-NMR ($CDCl_3$, 500 MHz), δ 7.30–7.26 (m, 1H), 7.24–7.17 (m, 8H), 7.09–7.05 (m, 4H), 7.01–6.98 (m, 1H), 6.93–6.91 (m, 2H), 6.89–6.87 (m, 1H), 6.66–6.63 (m, 1H), 6.17 (d, 1H, *J* = 5.00 Hz), 5.85 (s, 1H), 4.56 (s, 2H), 3.57–3.49 (m, 3H), 3.19–3.13 (m, 1H), 3.04, 2.85 (Abq, 2H, *J* = 10.00 Hz), 2.18–2.07 (m, 2H). ^{13}C-NMR ($CDCl_3$, 125 MHz), δ 170.17 (C), 150.58 (C), 139.35 (C), 137.05 (C), 134.59 (C), 131.75 (C), 130.14 (CH × 2), 128.77 (CH × 2), 128.63 (CH × 2), 128.56 (CH), 128.25 (CH × 2), 128.08 (CH × 2), 126.95

(CH × 2), 126.71 (CH), 126.60 (CH), 126.50 (CH), 123.15 (CH), 117.16 (CH), 106.54 (CH), 83.22 (CH), 56.42 (C), 50.30 (CH_2), 46.62 (CH_2), 44.62 (CH_2), 37.46 (CH_2), 25.61 (CH_2). ESI-MS: $[M + Na]^+$ Found 458.24.

1-(3a,8-Dibenzyl-3,3a,8,8a-tetrahydropyrrolo[2,3-b]indol-1(2H)-yl)-3-phenylpropan-1-one (**a11**): Reagents: benzene propanoic acid. Orange oily liquid, $[a]^{25}_{D}$ = −7.622, petroleum ether:ethyl acetate = 3:1, 88% Yield, ^{1}H-NMR ($CDCl_3$, 500 MHz), δ 7.23–7.14 (m, 11H), 7.08–7.07 (m, 2H), 7.02–6.99 (m, 1H), 6.92–6.91 (m, 2H), 6.87–6.85 (m, 1H), 6.65–6.62 (m, 1H), 6.20 (d, 1H, *J* = 5.00 Hz), 5.80 (s, 1H), 4.60, 4.51 (Abq, 2H, *J* = 15.00 Hz), 3.40–3.36 (m, 1H), 3.15–3.09 (m, 1H), 3.02(m, 1H), 2.83(m, 3H), 2.49–2.42 (m, 1H), 2.39–2.30 (m, 1H), 2.15–2.12(m, 1H), 2.06–2.00 (m, 1H). ^{13}C-NMR ($CDCl_3$, 125 MHz), δ 171.54 (C), 150.50 (C), 141.19 (C), 139.45 (C), 137.11 (C), 131.71 (C), 130.15 (CH × 2), 128.56 (CH), 128.48 (CH × 2), 128.39 (CH × 2), 128.25 (CH × 2), 128.07 (CH × 2), 126.99 (CH × 2), 126.58 (CH), 126.54 (CH), 126.11 (CH), 123.17 (CH), 117.11 (CH), 106.48 (CH), 83.85 (CH), 56.31 (C), 50.03 (CH_2), 46.35 (CH_2), 44.51 (CH_2), 37.28 (CH_2), 36.68 (CH_2), 31.07 (CH_2). ESI-MS: $[M + Na]^+$ Found 472.25.

(3a,8-Dibenzyl-3,3a,8,8a-tetrahydropyrrolo[2,3-b]indol-1(2H)-yl)(4-fluorophenyl)methanone (**a12**): Reagents: *p*-fluorobenzoic acid. White powder, mp 146–148 °C, $[a]^{25}_{D}$ = +70.611, petroleum ether:ethyl acetate = 4:1, 85% Yield, ^{1}H-NMR ($CDCl_3$, 500 MHz), δ 7.40–7.37 (m, 2H), 7.48–7.45 (m, 2H), 7.23–7.15 (m, 6H), 7.17–7.14 (m, 2H), 7.05–7.00 (m, 5H), 6.95–6.94 (m, 2H), 6.89–6.87 (m, 1H), 6.06 (s, 1H), 4.65, 4.48 (Abq, 2H, *J* = 15.00 Hz), 3.52–3.46 (m, 1H), 3.37–3.32 (m, 1H), 3.12, 2.93 (Abq, 2H, *J* = 15.00 Hz), 2.20–2.09 (m, 2H). ^{13}C-NMR ($CDCl_3$, 125 MHz), δ 169.43 (C), 163.79 (C) ($^1J_{C-F}$ = 247.50 Hz), 150.76 (C), 139.19 (C), 137.12 (C), 132.13 (C) ($^4J_{C-C-C-C-F}$ = 2.5 Hz), 131.22 (C), 130.13 (CH × 2), 129.86 (CH × 2), (d, $^3J_{C-C-C-F}$ = 8.75 Hz), 128.70 (CH), 128.32 (CH × 2), 128.08 (CH × 2), 126.81 (CH × 2), 126.60 (CH × 2), 123.48 (CH), 117.23 (CH), 115.22 (CH × 2), (d, $^2J_{C-C-F}$ = 21.25 Hz), 106.36 (CH), 82.75 (CH), 56.61 (C), 49.54 (CH_2), 49.54 (CH_2), 44.91 (CH_2), 37.90 (CH_2). ESI-MS: $[M + Na]^+$ Found 462.21.

(3-Chlorophenyl)(3a,8-dibenzyl-3,3a,8,8a-tetrahydropyrrolo[2,3-b]indol-1(2H)-yl)methanone (**a13**): Reagents: *m*-chlorobenzoic acid. Yellow solid15, mp 150–152 °C, $[a]^{25}_{D}$ = −15.523, petroleum ether:ethyl acetate = 3:1, 82% Yield, ^{1}H-NMR ($CDCl_3$, 500 MHz), δ 7.36–7.34 (m, 1H), 7.29–7.26 (m, 2H), 7.23–7.15 (m, 7H), 7.07–7.03 (m, 3H), 6.95–6.94 (m, 2H), 6.89–6.88 (m, 1H), 6.70–6.67 (m, 1H), 6.24 (d, 1H, *J* = 20.00 Hz), 6.04 (s, 1H), 4.65, 4.49 (Abq, 2H, *J* = 15.00 Hz), 3.48–3.44 (m, 1H), 3.35–3.30 (m, 1H), 3.14, 2.91 (Abq, 2H, *J* = 15.00 Hz), 2.20–2.09 (m, 2H). ^{13}C-NMR ($CDCl_3$, 125 MHz), δ 168.84 (C), 150.73 (C), 139.21 (C), 137.82 (C), 137.06 (C), 134.26 (C), 131.16 (C), 130.32 (CH), 130.12 (CH × 2), 129.55 (CH), 128.74 (CH), 128.35 (CH × 2), 128.10 (CH × 2), 127.59 (CH), 126.81 (CH × 2), 126.66 (CH), 126.64 (CH), 125.51 (CH), 123.49 (CH), 117.28 (CH), 106.31 (CH), 82.76 (CH), 56.65 (C), 49.53 (CH_2), 49.33 (CH_2), 44.90 (CH_2), 37.91 (CH_2). ESI-MS: $[M + Na]^+$ Found 478.18.

(4-Chlorophenyl)(3a,8-dibenzyl-3,3a,8,8a-tetrahydropyrrolo[2,3-b]indol-1(2H)-yl)methanone (**a14**): Reagents: *p*-chlorobenzoic acid. Yellow powder, mp 170–172 °C, $[a]^{25}_{D}$ = −13.183, petroleum ether:ethyl acetate = 3:1, 82% Yield, ^{1}H-NMR ($CDCl_3$, 500 MHz), δ 7.30 (s, 4H), 7.23–7.16 (m, 6H), 7.06–7.03 (m, 3H), 6.95–6.93 (m, 2H), 6.89–6.87 (m, 1H), 6.69–6.66 (m, 1H), 6.22 (d, 1H, *J* = 10.00 Hz), 6.05 (s, 1H), 4.65, 4.48 (Abq, 2H, *J* = 15.00 Hz), 3.49–3.45 (m, 1H), 3.36–3.30 (m, 1H), 3.12, 2.92 (Abq, 2H, *J* = 10.00 Hz), 2.20–2.09 (m, 2H). ^{13}C-NMR ($CDCl_3$, 125 MHz), δ 169.32 (C), 150.74 (C), 139.19 (C), 137.09 (C), 136.38 (C), 134.43 (C), 131.18 (C), 130.12 (CH × 2), 128.99 (CH × 2), 128.71 (CH), 128.43 (CH × 2), 128.33 (CH × 2), 128.08 (CH × 2), 126.81 (CH × 2), 126.61 (CH × 2), 123.48 (CH), 117.25 (CH), 106.35 (CH), 82.77 (CH), 56.63 (C), 49.55 (CH_2), 49.42 (CH_2), 44.91 (CH_2), 37.88 (CH_2). ESI-MS: $[M + Na]^+$ Found 478.18.

(3a,8-Dibenzyl-3,3a,8,8a-tetrahydropyrrolo[2,3-b]indol-1(2H)-yl)(o-tolyl)methanone (**a15**): Reagents: 2-methylbenzoic acid. Green powder, mp 142–144 °C, $[a]^{25}_{D}$ = −10.883, petroleum ether:ethyl acetate = 3:1, 85% Yield, ^{1}H-NMR ($CDCl_3$, 500 MHz), δ 7.23–7.18 (m, 6H), 7.15–7.07 (m, 5H), 7.04–6.98 (m, 3H), 6.92–6.91 (m, 2H), 6.68–6.65 (m, 1H), 6.21 (d, 1H), 6.06 (s, 1H), 4.73, 4.57 (Abq, 2H, *J* = 15.00 Hz), 3.22–3.18 (m, 1H), 3.15, 2.96 (Abq, 2H, *J* = 10.00 Hz), 3.12–3.07 (m, 1H), 2.15 (s, 3H), 2.12–2.09 (m, 2H). ^{13}C-NMR ($CDCl_3$, 125 MHz), δ 170.41 (C), 150.90 (C), 139.25 (C), 137.09 (C), 136.75 (C), 134.15 (C), 131.50 (C), 130.35 (CH), 130.20 (CH × 2), 128.95 (CH), 128.65 (CH), 128.32 (CH × 2), 128.14 (CH × 2), 126.91 (CH × 2), 126.62 (CH), 126.59 (CH), 125.85 (CH), 125.71 (CH), 123.40 (CH), 117.21 (CH), 106.16

(CH), 82.16 (CH), 57.07 (C), 49.78 (CH_2), 47.83 (CH_2), 45.01 (CH_2), 37.99 (CH_2), 18.87 (CH_3). ESI-MS: $[M + Na]^+$ Found 458.24.

(3a,8-Dibenzyl-3,3a,8,8a-tetrahydropyrrolo[2,3-b]indol-1(2H)-yl)(m-tolyl)methanone (**a16**): Reagents: m-toluic acid. Green solid, mp 134–136 °C, $[a]_D^{25}$ = −11.951, petroleum ether:ethyl acetate = 4:1, 85% Yield, ^{1}H-NMR ($CDCl_3$, 500 MHz), δ 7.22–7.15 (m, 10H), 7.06–7.02 (m, 3H), 6.94–6.94 (m, 2H) , 6.88–6.87 (m, 2H), 6.21 (d, 1H), 6.07 (s, 1H), 4.68, 4.48 (Abq, 2H, *J* = 15.00 Hz), 3.55–3.51 (m, 1H), 3.36–3.30 (m, 1H), 3.13, 2.93 (Abq, 2H, *J* = 15.00 Hz), 2.32 (s, 3H), 2.18–2.07 (m, 2H). ^{13}C-NMR ($CDCl_3$, 125 MHz), δ 170.71 (C), 150.88 (C), 139.25 (C), 137.21 (C), 136.05 (C), 134.35 (C), 130.98 (C), 130.67 (C), 130.14 (CH × 2), 128.63 (CH), 128.35 (CH), 128.30 (CH × 2), 128.05 (CH × 2), 127.32 (CH), 126.87 (CH × 2), 126.56 (CH), 126.54 (CH), 124.50 (CH), 123.49 (CH), 117.09 (CH), 106.26 (CH), 82.61 (CH), 56.66 (C), 49.47 (CH_2), 49.47 (CH_2), 44.99 (CH_2), 38.01 (CH_2), 21.28 (CH_3). ESI-MS: $[M + Na]^+$ Found 458.24.

(3a,8-Dibenzyl-3,3a,8,8a-tetrahydropyrrolo[2,3-b]indol-1(2H)-yl)(p-tolyl)methanone (**a17**): Reagents: 4-methylbenzoic acid. White powder, mp 120–122 °C, $[a]_D^{25}$ = −9.018, petroleum ether:ethyl acetate = 4:1, 87% Yield, ^{1}H-NMR ($CDCl_3$, 500 MHz), δ 7.31–7.30 (m, 2H), 7.20–7.13 (m, 8H), 7.04–7.01 (m, 3H), 6.95–6.94 (m, 2H), 6.88–6.86 (m, 1H), 6.68–6.65 (m, 1H), 6.18 (d, 1H), 6.08 (s, 1H), 4.68, 4.47 (Abq, 2H, *J* = 20.00 Hz), 3.57–3.54 (m, 1H), 3.38–3.32 (m, 1H), 3.13, 2.93 (Abq, 2H, *J* = 15.00 Hz), 2.35 (s, 3H), 2.18–2.07 (m, 2H). ^{13}C-NMR ($CDCl_3$, 125 MHz), δ 170.71 (C), 150.88 (C), 139.25 (C), 137.21 (C), 136.05 (C), 134.35 (C), 130.98 (CH), 130.14 (CH × 2), 128.75 (CH × 2), 128.61 (CH), 128.28 (CH × 2), 128.04 (CH × 2), 127.67 (CH × 2), 126.83 (CH × 2), 126.54 (CH), 126.52 (CH), 123.47 (CH), 117.08 (CH), 106.31 (CH), 82.66 (CH), 56.62 (C), 49.55 (CH_2), 49.49 (CH_2), 44.98 (CH_2), 37.99 (CH_2), 21.40 (CH_3). ESI-MS: $[M + Na]^+$ Found 458.24.

(3a,8-Dibenzyl-3,3a,8,8a-tetrahydropyrrolo[2,3-b]indol-1(2H)-yl)(4-methoxyphenyl)methanone (**a18**): Reagents: *p*-methoxyl benzoic acid. Orange oily liquid, $[a]_D^{25}$ = −17.999, petroleum ether:ethyl acetate = 5:1, 83% Yield, ^{1}H-NMR ($CDCl_3$, 500 MHz), δ 7.21–7.16 (m, 6H), 7.06–7.05 (m, 2H), 6.95–6.94 (m, 2H), 7.00–6.97 (m, 3H), 6.93–6.6.91 (m, 2H), 6.88–6.87 (m, 1H), 6.79–6.77 (m, 2H), 6.65–6.62 (m, 1H), 6.16 (d, 1H), 5.84 (s, 1H), 4.55 (s, 2H), 3.77 (s, 3H), 3.57–3.52 (m, 1H), 3.18–3.13 (m, 1H), 3.04, 2.85 (Abq, 2H, *J* = 15.00 Hz), 2.17–2.06 (m, 2H). ^{13}C-NMR ($CDCl_3$, 125 MHz), δ 170.52 (C), 158.37 (C), 150.59 (C), 139.39 (C), 137.09 (C), 131.80 (C), 130.15 (CH × 2), 129.80 (CH), 128.55 (CH), 128.07 (CH × 2), 128.24 (CH × 2), 128.07 (CH × 2), 126.98 (CH × 2), 126.59 (CH), 126.49 (CH), 123.15 (CH), 117.16 (CH), 114.08 (CH × 2), 106.55 (CH), 83.26 (CH), 56.40 (C), 55.24 (CH_3), 50.32 (CH_2), 44.63 (CH_2), 41.32 (CH_2), 37.50 (CH_2). ESI-MS: $[M + Na]^+$ Found 474.23.

3a,8-Dibenzyl-1-(methylsulfonyl)-1,2,3,3a,8,8a-hexahydropyrrolo[2,3-b]indole (**a19**): Reagents: mesyl chloride. Yellow solid, mp 148–150 °C, $[a]_D^{25}$ = −16.158, petroleum ether:ethyl acetate = 5:1, 87% Yield, ^{1}H-NMR ($CDCl_3$, 500 MHz), δ 7.29–7.26 (m, 2H), 7.23–7.22 (m, 3H), 7.17–7.15 (m, 2H), 7.09–7.06 (m, 1H), 6.98–6.96 (m, 1H), 6.91–6.89 (m, 2H), 6.73–6.70 (m, 1H), 6.36 (d, 1H), 5.33 (s, 1H), 4.61, 4.47 (Abq, 2H, *J* = 15.00 Hz), 3.60–3.57 (m, 1H), 3.08–3.01 (m, 1H), 2.93, 2.89 (Abq, 2H, *J* = 15.00 Hz), 2.31 (s, 3H), 2.27–2.16 (m, 2H), 1.28–1.24 (m, 1H). ^{13}C-NMR ($CDCl_3$, 125 MHz), δ 150.10 (C), 138.61 (C), 136.73 (C), 131.54 (C), 130.53 (CH × 2), 128.91 (CH), 128.52 (CH × 2), 128.38 (CH × 2), 127.31 (CH × 2), 127.05 (CH), 126.88 (CH), 123.21 (CH), 117.86 (CH), 106.72 (CH), 85.04 (CH), 58.69 (C), 48.47 (CH_2), 47.56 (CH_2), 43.94 (CH_2), 37.95 (CH_2), 37.03 (CH_3). ESI-MS: $[M + Na]^+$ Found 45817.

3a,8-Dibenzyl-1-(phenylsulfonyl)-1,2,3,3a,8,8a-hexahydropyrrolo[2,3-b]indole (**a20**): Reagents: benzene sulfonyl chloride. Green solid, mp 158–160 °C, $[a]_D^{25}$ = −14.675, petroleum ether:ethyl acetate = 4:1, 91% Yield, ^{1}H-NMR ($CDCl_3$, 500 MHz), δ 7.71–7.70 (m, 2H), 7.59–7.56 (m, 1H), 7.48–7.45 (m, 2H), 7.24–7.20 (m, 4H), 7.18–7.15 (m, 2H), 7.02–6.99 (m, 1H), 6.96–6.94 (m, 2H), 6.88–6.86 (m, 1H), 6.77–6.75 (m, 2H), 6.66–6.64 (m, 1H), 6.18 (d, 1H,), 5.43 (s, 1H), 4.77, 4.39 (Abq, 2H, *J* = 15.00 Hz), 3.61–3.57 (m, 1H), 3.09–3.03 (m, 1H), 2.90, 2.53 (Abq, 2H, *J* = 15.00 Hz), 1.97–1.94 (m, 1H), 1.52–1.46 (m, 1H). ^{13}C-NMR ($CDCl_3$, 125 MHz), δ 150.22 (C), 139.71 (C), 138.47 (C), 136.92 (C), 132.71 (C), 130.67 (CH), 130.00 (CH × 2), 129.25 (CH × 2), 128.76 (CH), 128.40 (CH × 2), 128.26 (CH × 2), 127.15 (CH × 2), 126.90 (CH × 2), 126.72 (CH), 126.66 (CH), 123.10 (CH), 117.40 (CH), 106.27 (CH), 86.37 (CH), 58.57 (C), 47.97 (CH_2), 47.72 (CH_2), 44.34 (CH_2), 38.05 (CH_2). ESI-MS: $[M + Na]^+$ Found 480.19.

3a,8-Dibenzyl-1-tosyl-1,2,3,3a,8,8a-hexahydropyrrolo[2,3-b]indole (**a21**): Reagents: *p*-toluensulfonyl chloride. Yellow solid, mp 164–166 °C, $[a]_D^{25}$ = −16.158, petroleum ether:ethyl acetate = 3:1, 95% Yield, ^{1}H-NMR ($CDCl_3$, 500 MHz), δ 7.60 (d, 2H, *J* = 10.00 Hz), 7.26 (m, 1H), 7.48–7.45 (m, 2H), 7.24–7.18 (m, 4H), 7.17–7.14 (m, 2H), 7.01–6.98 (m, 1H), 6.94–6.92 (m, 2H), 6.88–6.87 (m, 1H), 6.77–6.76 (m, 2H), 6.66–6.63 (m, 1H), 6.16 (d, 2H, *J* = 20.00 Hz), 5.41 (s, 1H), 4.77, 4.37 (Abq, 2H, *J* = 15.00 Hz), 3.60–3.57 (m, 1H), 3.08–3.02 (m, 1H), 2.92, 2.52 (Abq, 2H, *J* = 10.00 Hz), 1.96–1.93 (m, 1H), 1.53–1.46 (m, 1H). ^{13}C-NMR ($CDCl_3$, 125 MHz), δ 150.28 (C), 143.51 (C), 138.51 (C), 136.99 (C), 136.76 (C), 130.71 (C), 130.00 (CH × 2), 129.84 (CH × 2), 128.73 (CH), 128.37 (CH × 2), 128.22 (CH × 2), 127.20 (CH × 2), 126.87 (CH × 2), 126.66 (CH), 126.60 (CH), 123.07 (CH), 117.33 (CH), 106.22 (CH), 86.32 (CH), 58.58 (C), 47.99 (CH_2), 47.68 (CH_2), 44.38 (CH_2), 38.15 (CH_2), 21.56 (CH_3). ESI-MS: $[M + Na]^+$ Found 494.20.

1-(3a,8-Diethyl-3,3a,8,8a-tetrahydropyrrolo[2,3-b]indol-1(2H)-yl)ethan-1-one (**b1**):Reagents: acetic anhydride. Picine oil, $[a]_D^{25}$ = −14.549, petroleum ether:ethyl acetate = 3:1, 94% Yield, ^{1}H-NMR ($CDCl_3$, 500 MHz), δ 7.10–7.07 (m, 1H), 6.97–6.95 (m, 1H), 6.65–6.62 (m, 1H), 6.36 (d, 1H, *J*=5.00 Hz), 5.64 (s, 1H), 3.63–3.59 (m, 1H), 3.56–3.47 (m, 2H), 3.29–3.23 (m, 1H), 2.14–2.10 (m, 1H), 2.07 (s, 3H), 2.01–1.95 (m, 1H), 1.84–1.76 (m, 1H), 1.73–1.65 (m, 1H), 1.14 (t, 3H, *J*=10.00 Hz), 0.82 (t, 3H, *J*=10.00 Hz). ^{13}C-NMR ($CDCl_3$, 125 MHz), δ 169.73 (C), 150.23 (C), 132.05 (C), 128.32 (CH), 122.79 (CH), 116.67 (CH), 105.39 (CH), 81.98 (CH), 55.90 (C), 46.86 (CH_2), 40.14 (CH_2), 38.34 (CH_2), 31.87 (CH_2), 22.87 (CH_3), 12.39 (CH_3), 9.41 (CH_3). ESI-MS: $[M + Na]^+$ Found 258.17.

1-(3a,8-Diethyl-3,3a,8,8a-tetrahydropyrrolo[2,3-b]indol-1(2H)-yl)propan-1-one (**b2**): Reagents: propionic andydride. Picine oil, petroleum ether:ethyl acetate = 3:1, 94% Yield, ^{1}H-NMR ($CDCl_3$, 500 MHz), δ 7.10–7.07 (m, 1H), 6.97–6.95 (m, 1H), 6.64–6.61 (m, 1H), 6.36 (d, 1H, *J* = 5.00 Hz), 5.66 (s, 1H), 3.66–3.62 (m, 1H), 3.57–3.46 (m, 2H), 3.25–3.20 (m, 1H), 2.38–2.23 (m, 2H), 2.14–2.10 (m, 1H), 1.99–1.93 (m, 1H), 2.01–1.95 (m, 1H), 1.84–1.76 (m, 1H), 1.72–1.65 (m, 1H), 1.16 (t, 3H, *J*=5.00 Hz), 1.13(t, 3H, *J*=5.00 Hz), 0.82 (t, 3H, *J*=10.00 Hz).^{13}C-NMR ($CDCl_3$, 125 MHz), δ 173.05 (C), 150.24 (C), 132.12 (C), 128.29 (CH), 122.78 (CH), 116.62 (CH), 105.36 (CH), 82.15 (CH), 55.64 (C), 45.90 (CH_2), 40.15 (CH_2), 38.39 (CH_2), 31.85 (CH_2), 28.11 (CH_2), 12.33 (CH_3), 9.42 (CH_3), 9.08 (CH_3). ESI-MS: $[M + Na]^+$ Found 272.19.

1-(3a,8-Diethyl-3,3a,8,8a-tetrahydropyrrolo[2,3-b]indol-1(2H)-yl)butan-1-one (**b3**): Reagents: butyric anhydride. Picine oil, petroleum ether:ethyl acetate = 4:1, 91% Yield, ^{1}H-NMR ($CDCl_3$, 500 MHz), δ 7.10–7.07 (m, 1H), 6.97–6.95 (m, 1H), 6.64–6.61 (m, 1H), 6.36 (d, 1H, *J* = 5.00 Hz), 5.67 (s, 1H), 3.67–3.63 (m, 1H), 3.55–3.47 (m, 2H), 3.26–3.20 (m, 1H), 2.34–2.21 (m, 2H), 2.14–2.10 (m, 1H), 1.99–1.92 (m, 1H), 1.84–1.76 (m, 1H), 1.72–1.64 (m, 3H), 1.14 (t, 3H, *J* = 5.00 Hz), 0.97(t, 3H, *J*=5.00 Hz), 0.81 (t, 3H, *J* = 5.00 Hz).^{13}C-NMR ($CDCl_3$, 125 MHz), δ 172.38 (C), 150.28 (C), 132.11 (C), 128.29 (CH), 122.79 (CH), 116.62 (CH), 105.32 (CH), 82.07 (CH), 55.65 (C), 46.08 (CH_2), 40.16 (CH_2), 38.44 (CH_2), 36.90 (CH_2), 31.90 (CH_2), 18.39 (CH_3), 12.39 (CH_3), 9.42 (CH_3). ESI-MS: $[M + Na]^+$ Found 286.20.

1-(3a,8-Diethyl-3,3a,8,8a-tetrahydropyrrolo[2,3-b]indol-1(2H)-yl)-2-methylpropan-1-one (**b4**): Reagents: isobutyric anhydride. Picine oil, petroleum ether:ethyl acetate = 4:1, 92% Yield, ^{1}H-NMR ($CDCl_3$, 500 MHz), δ 7.10–7.07 (m, 1H), 6.97–6.96 (m, 1H), 6.64–6.61 (m, 1H), 6.36 (d, 1H, *J* = 5.00 Hz), 5.69 (s, 1H), 3.74–3.70 (m, 1H), 3.53–3.45 (m, 2H), 3.27–3.22 (m, 1H), 2.69–2.57 (m, 1H), 2.15–2.11 (m, 1H), 1.99–1.93 (m, 1H), 1.84–1.77 (m, 1H), 1.73–1.66 (m, 3H), 1.15–1.12 (m, 9H), 0.81(t, 3H, *J* = 5.00 Hz).^{13}C-NMR ($CDCl_3$, 125 MHz), δ 176.27 (C), 150.39 (C), 132.06 (C), 128.29 (CH), 122.82 (CH), 116.54 (CH), 105.10 (CH), 82.19 (CH), 55.53 (C), 45.77 (CH_2), 40.13 (CH_2), 38.66 (CH_2), 32.25 (CH_2), 32.06 (CH_2), 19.22 (CH_3), 18.83 (CH_3), 12.52 (CH_3), 9.41 (CH_3). ESI-MS: $[M + Na]^+$ Found 286.20.

1-(3a,8-Dipropyl-3,3a,8,8a-tetrahydropyrrolo[2,3-b]indol-1(2H)-yl)ethan-1-one (**c1**): Reagents: acetic anhydride. Picine oil, petroleum ether:ethyl acetate = 3:1, 93% Yield, ^{1}H-NMR ($CDCl_3$, 500 MHz), δ 7.08–7.05 (m, 1H), 6.96–6.95 (m, 1H), 6.63–6.60 (m, 1H), 6.34 (d, 1H, *J* = 5.00 Hz), 5.65 (s, 1H), 3.62–3.58 (m, 1H), 3.43–3.38 (m, 1H), 3.35–3.29 (m, 1H), 3.25–3.20 (m, 1H), 2.14–2.10 (m, 1H), 2.07 (s, 3H), 2.00–1.94 (m, 1H), 1.76–1.52 (m, 4H), 1.38–1.22 (m, 1H), 1.16–1.06 (m, 1H), 0.91(t, 3H, *J*=5.00 Hz). 0.85(t, 3H, *J* = 5.00 Hz).^{13}C-NMR ($CDCl_3$, 125 MHz), δ 169.73 (C), 150.67 (C), 132.12 (C), 128.25 (CH), 122.72 (CH), 116.48 (CH), 105.14 (CH), 82.70 (CH), 55.52 (C), 47.50 (CH_2), 46.80 (CH_2), 41.71 (CH_2), 38.84 (CH_2), 22.86 (CH_3), 20.85 (CH_2), 18.41(CH_2), 14.48 (CH_3), 11.57 (CH_3). ESI-MS: $[M + Na]^+$ Found 286.20.

1-(3a,8-Dipropyl-3,3a,8,8a-tetrahydropyrrolo[2,3-b]indol-1(2H)-yl)propan-1-one (**c2**): Reagents: propionic andydride. Picine oil, petroleum ether:ethyl acetate = 3:1, 91% Yield, ^{1}H-NMR ($CDCl_3$, 500 MHz), δ 7.08–7.05 (m, 1H), 6.96–6.95 (m, 1H), 6.62–6.59 (m, 1H), 6.34 (d, 1H, *J* = 5.00 Hz), 5.67 (s, 1H), 3.65–3.61 (m, 1H), 3.45–3.39 (m, 1H), 3.36–3.30 (m, 1H), 3.21–3.16 (m, 1H), 2.38–2.22 (m, 2H), 2.14–2.10 (m, 1H), 1.98–1.92 (m, 1H), 1.75–1.52 (m, 5H), 1.38–1.28 (m, 1H), 1.15 (t, 3H, *J* = 5.00 Hz), 0.91(t, 3H, *J* = 5.00 Hz). 0.85(t, 3H, *J* = 5.00 Hz).^{13}C-NMR ($CDCl_3$, 125 MHz), δ 173.03 (C), 150.72 (C), 132.21 (C), 128.23 (CH), 122.73 (CH), 116.43 (CH), 105.15 (CH), 82.90 (CH), 55.31 (C), 47.48 (CH_2), 45.84 (CH_2), 41.76 (CH_2), 38.88 (CH_2), 28.15 (CH_2), 20.87 (CH_2), 18.43(CH_2), 14.48 (CH_3), 11.58 (CH_3) , 9.17 (CH_3). ESI-MS: $[M + Na]^+$ Found 300.22.

1-(3a,8-Dipropyl-3,3a,8,8a-tetrahydropyrrolo[2,3-b]indol-1(2H)-yl)butan-1-one (**c3**): Reagents: butyric anhydride. Picine oil, petroleum ether:ethyl acetate = 4:1, 92% Yield, ^{1}H-NMR ($CDCl_3$, 500 MHz), δ 7.08–7.05 (m, 1H), 6.96–6.94 (m, 1H), 6.62–6.59 (m, 1H), 6.34 (d, 1H, *J* = 5.00 Hz), 5.68 (s, 1H), 3.66–3.62 (m, 1H), 3.43–3.30 (m, 2H), 3.21–3.16 (m, 1H), 2.34–2.20 (m, 2H), 2.13–2.09 (m, 1H), 1.97–1.91 (m, 1H), 1.77–1.52 (m, 6H), 1.38–1.27 (m, 1H), 1.17–1.06 (m, 1H), 0.97 (t, 3H, *J* = 5.00 Hz), 0.90(t, 3H, *J* = 5.00 Hz). 0.85(t, 3H, *J* = 5.00 Hz).^{13}C-NMR ($CDCl_3$, 125 MHz), δ 172.35 (C), 150.77 (C), 132.18 (C), 128.23 (CH), 122.74 (CH), 116.41 (CH), 105.08 (CH), 82.77 (CH), 55.31 (C), 47.45 (CH_2), 46.02 (CH_2), 41.80 (CH_2), 38.93 (CH_2), 36.93 (CH_2), 28.15 (CH_2), 20.89 (CH_2), 18.46 (CH_2), 18.42 (CH_2), 14.48 (CH_3), 13.94 (CH_3) , 11.58 (CH_3). ESI-MS: $[M + Na]^+$ Found 314.24.

1-(3a,8-Dipropyl-3,3a,8,8a-tetrahydropyrrolo[2,3-b]indol-1(2H)-yl)-2-methylpropan-1-one (**c4**): Reagents: isobutyric anhydride. Picine oil, petroleum ether:ethyl acetate = 5:1, 93% Yield, ^{1}H-NMR ($CDCl_3$, 500 MHz), δ 7.08–7.05 (m, 1H), 6.96–6.95 (m, 1H), 6.62–6.59 (m, 1H), 6.33 (d, 1H, *J* = 5.00 Hz), 5.69 (s, 1H), 3.73–3.69 (m, 1H), 3.40–3.30 (m, 2H), 3.23–3.17 (m, 1H), 2.70–2.62 (m, 1H), 2.14–2.10 (m, 1H), 1.97–1.91(m, 1H), 1.76–1.49 (m, 5H), 1.36–1.29 (m, 1H), 1.13 (t, 6H, *J* = 5.00 Hz), 0.90(t, 3H, *J* = 5.00 Hz). 0.85(t, 3H, *J* = 5.00 Hz).^{13}C-NMR ($CDCl_3$, 125 MHz), δ 176.16 (C), 150.88 (C), 132.20 (C), 128.22 (CH), 122.77 (CH), 116.39 (CH), 105.00 (CH), 82.88 (CH), 55.21 (C), 47.42 (CH_2), 45.69 (CH_2), 41.95 (CH_2), 39.16 (CH_2), 32.25 (CH_3), 21.00 (CH_2), 19.30 (CH_3), 18.83 (CH_3), 18.43 (CH_2), 14.47 (CH_3), 11.59 (CH_3). ESI-MS: $[M + Na]^+$ Found 314.24.

2. ^{1}H- and ^{13}C-NMR Spectra

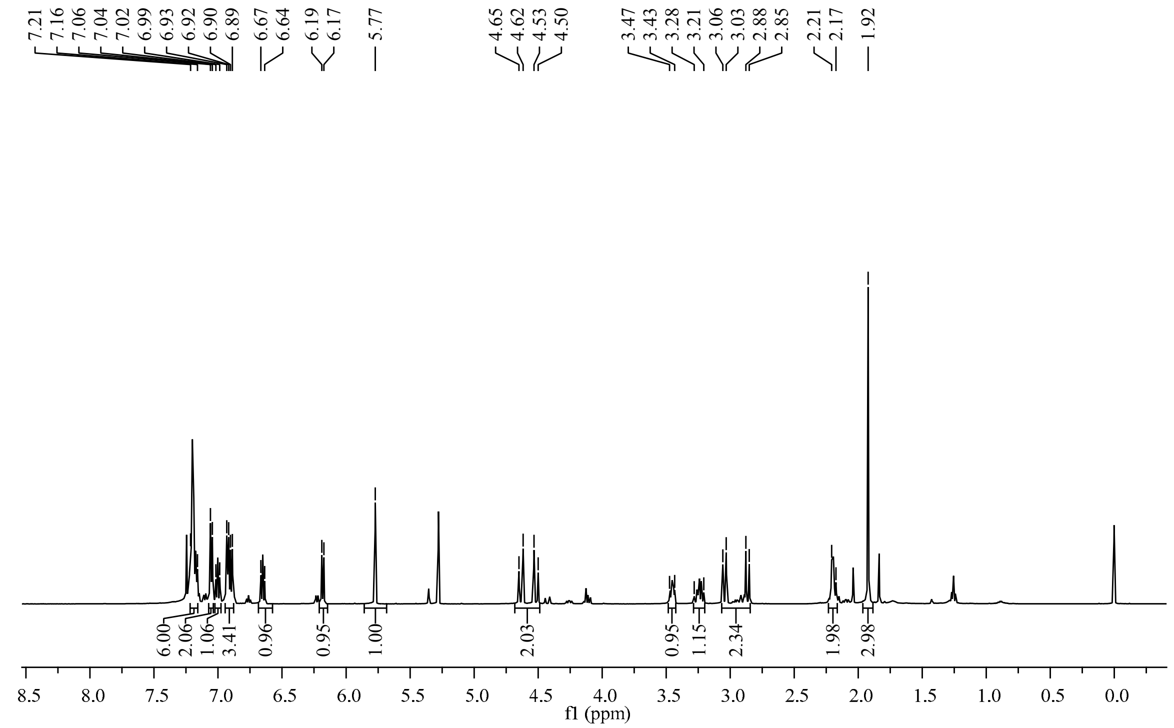

Figure S1. ^{1}H-NMR spectroscopic data for compound **a1**.

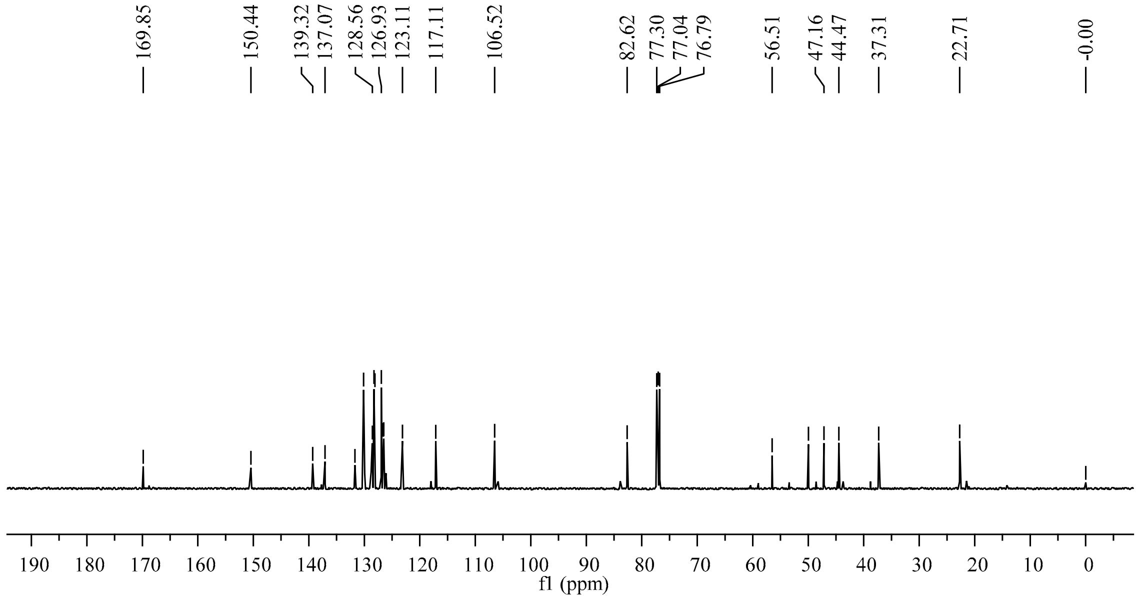

Figure S2. ^{13}C-NMR spectroscopic data for compound **a1**.

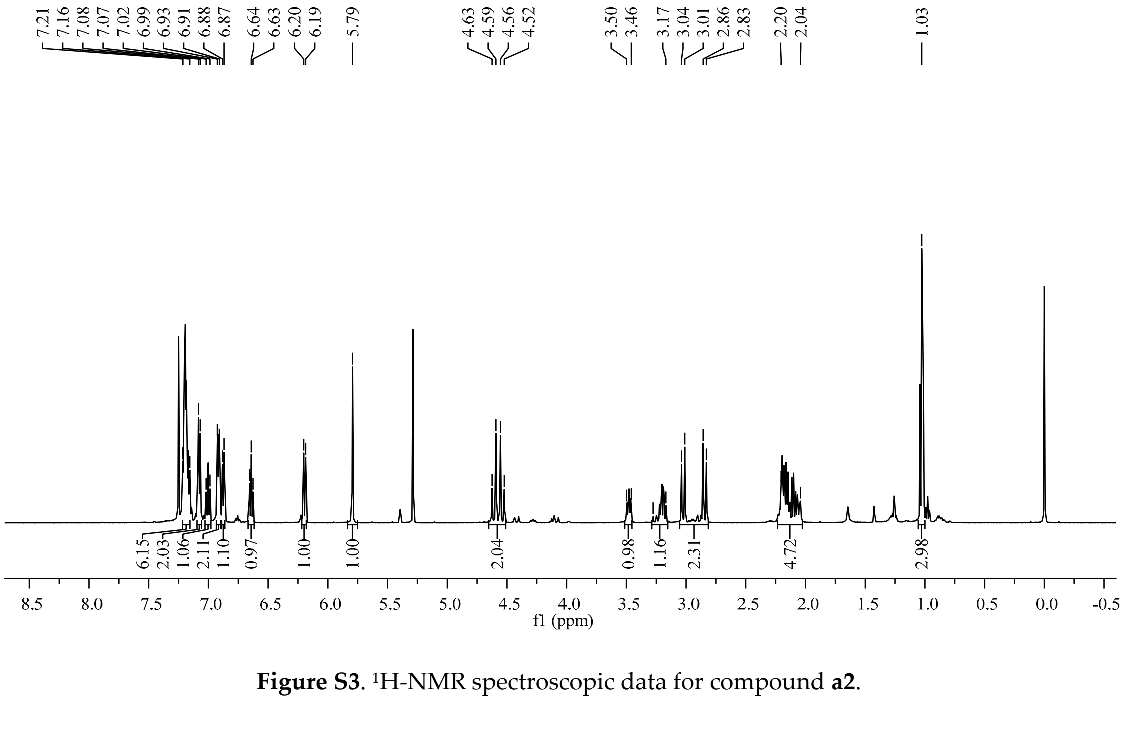

Figure S3. ^{1}H-NMR spectroscopic data for compound **a2**.

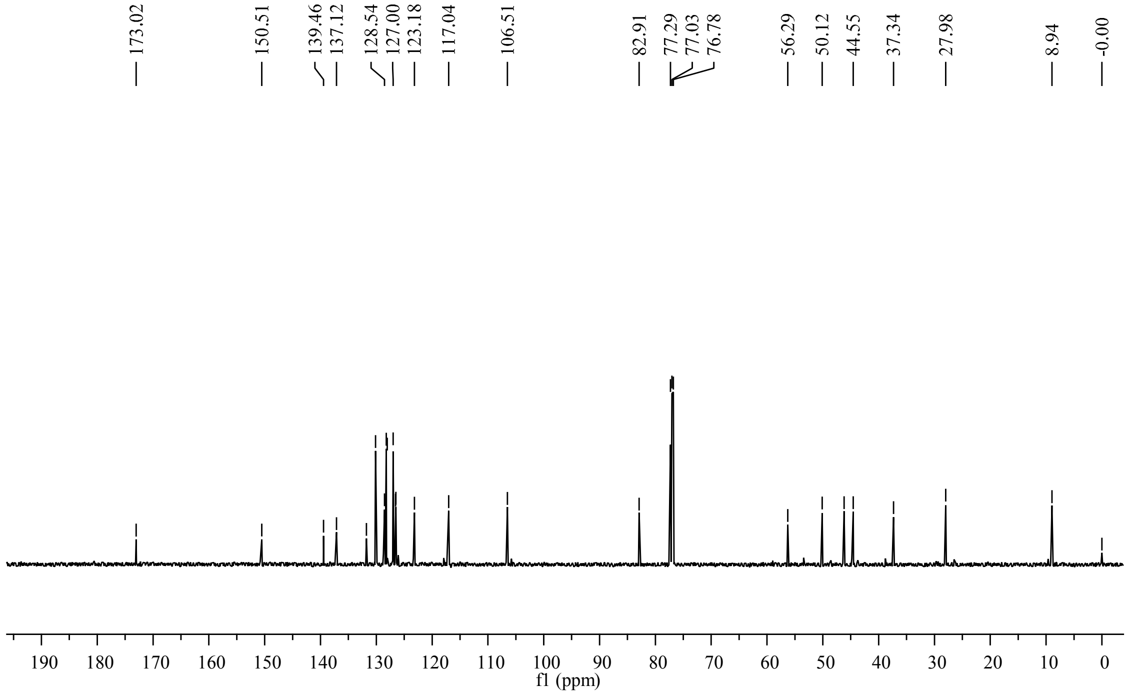

Figure S4. ^{13}C-NMR spectroscopic data for compound **a2**.

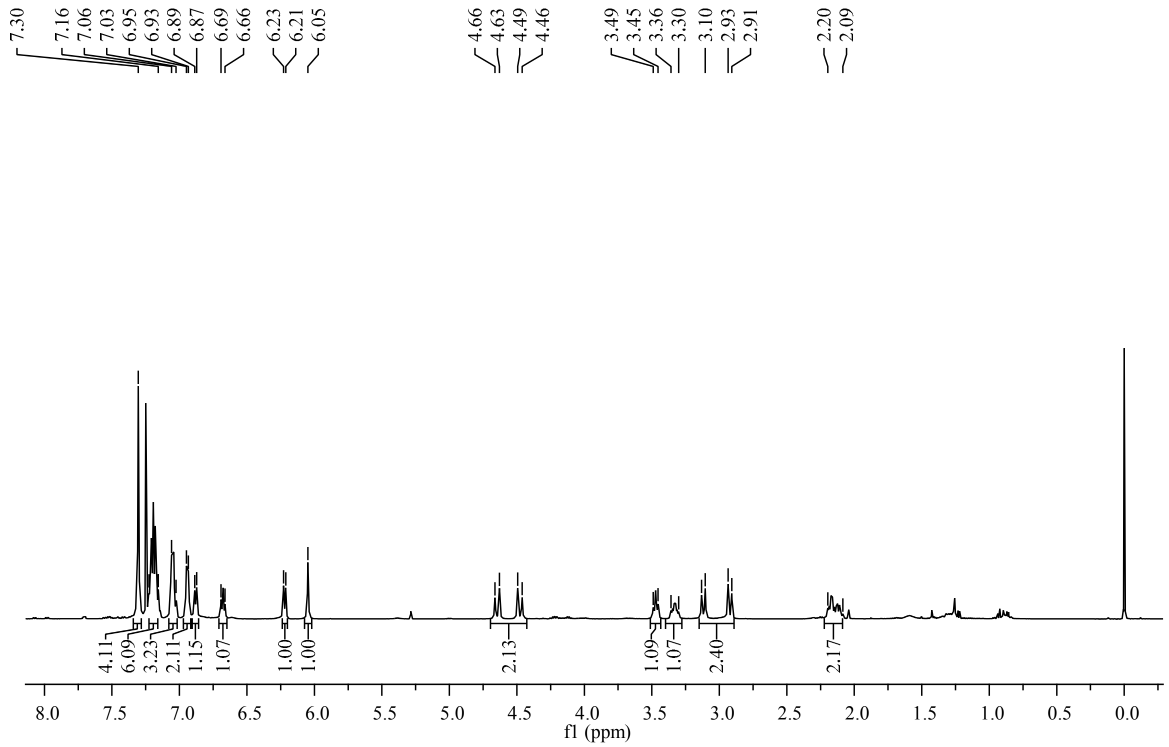

Figure S5. ^{1}H-NMR spectroscopic data for compound **a14**.

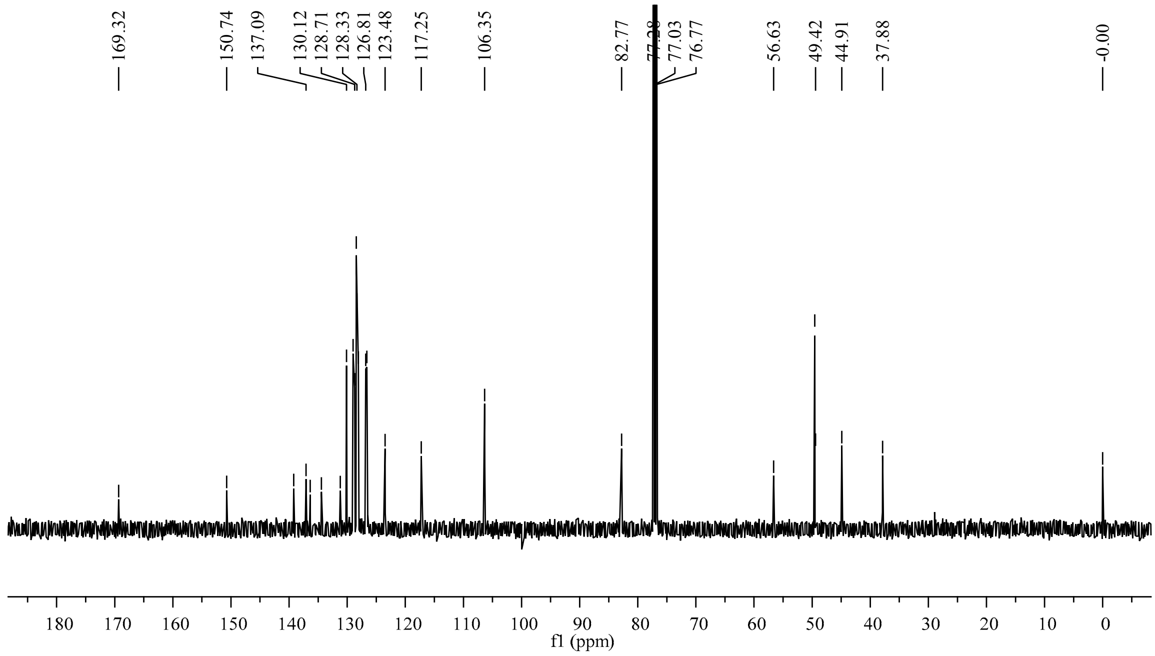

Figure S6. ^{13}C-NMR spectroscopic data for compound **a14**.

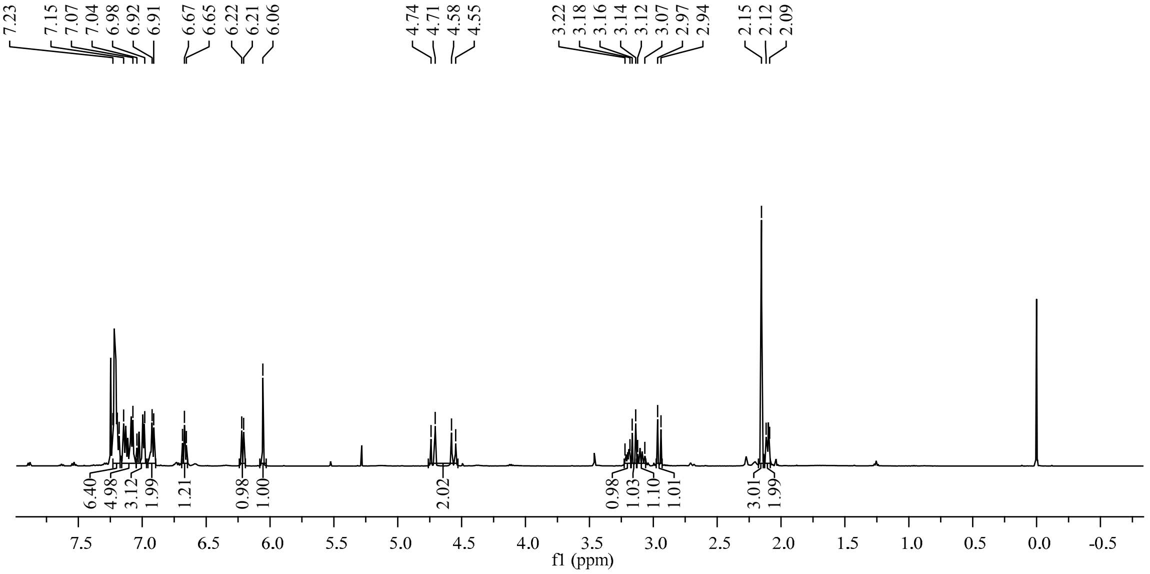

Figure S7. ^{1}H-NMR spectroscopic data for compound **a15**.

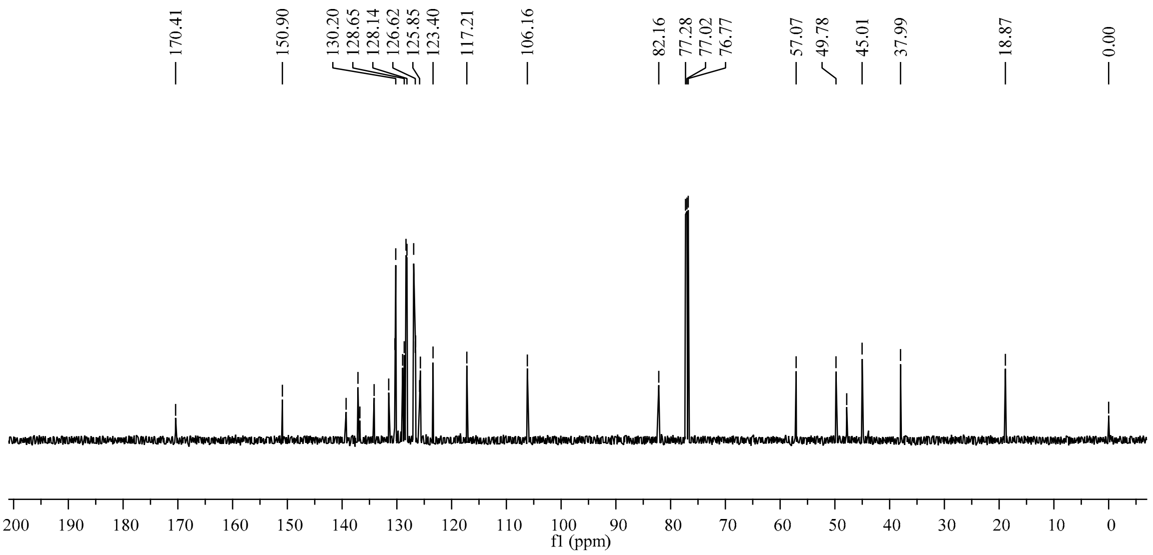

Figure S8. ^{13}C-NMR spectroscopic data for compound **a15**.

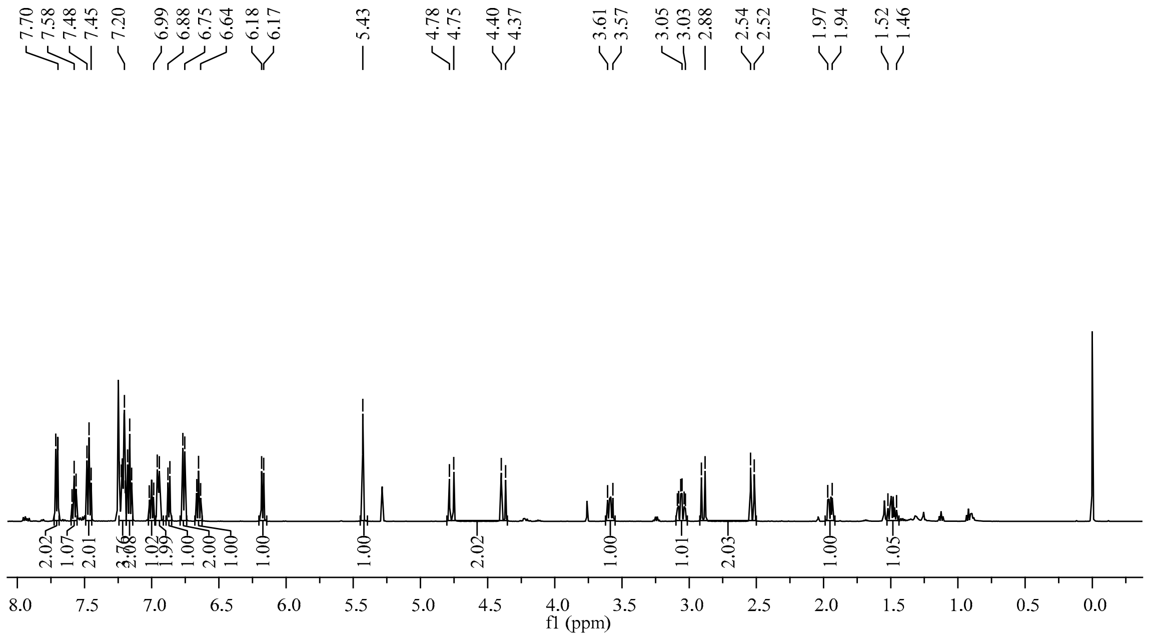

Figure S9. ^{1}H-NMR spectroscopic data for compound **a20**.

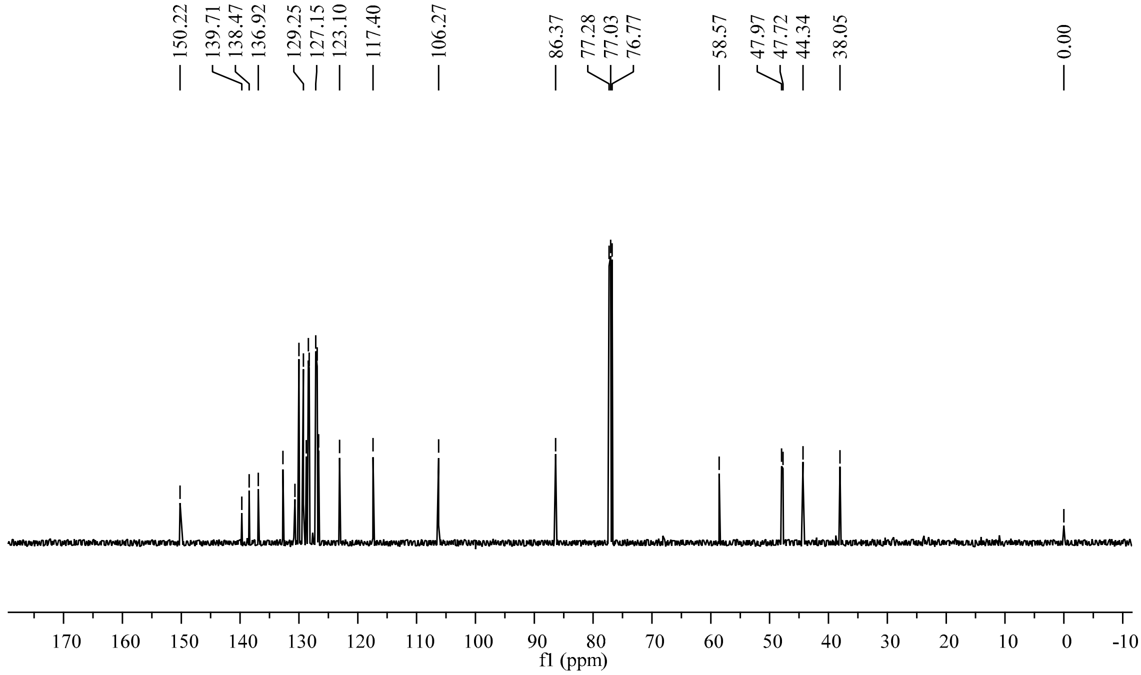

Figure S10. ^{13}C-NMR spectroscopic data for compound **a20**

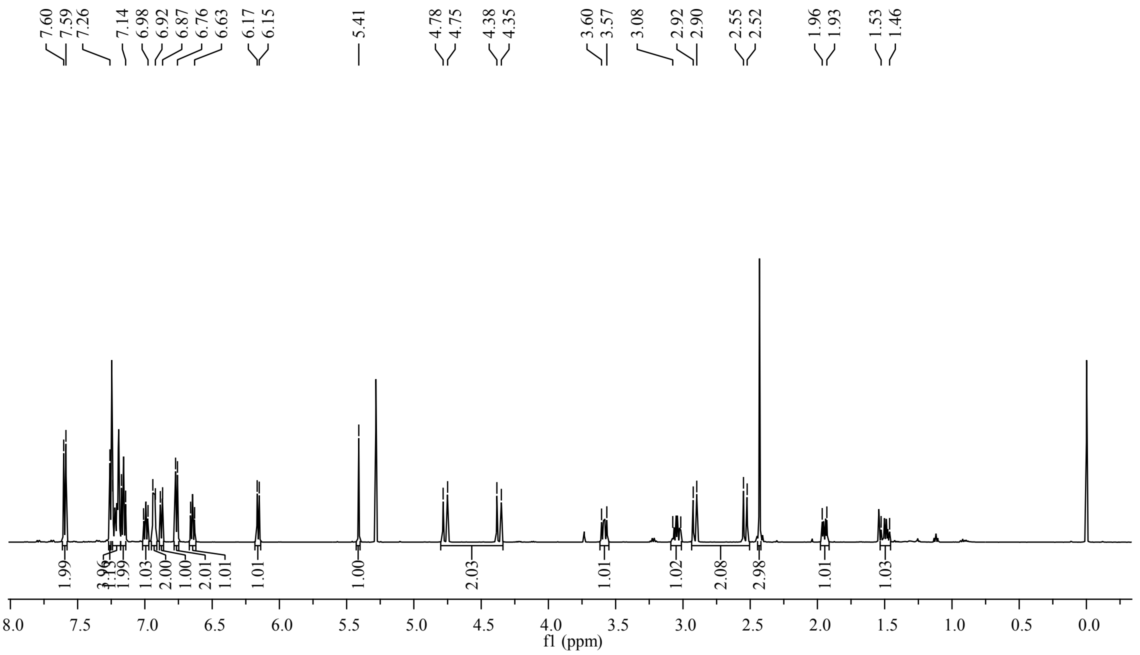

Figure S11. ^{1}H-NMR spectroscopic data for compound **a21**.

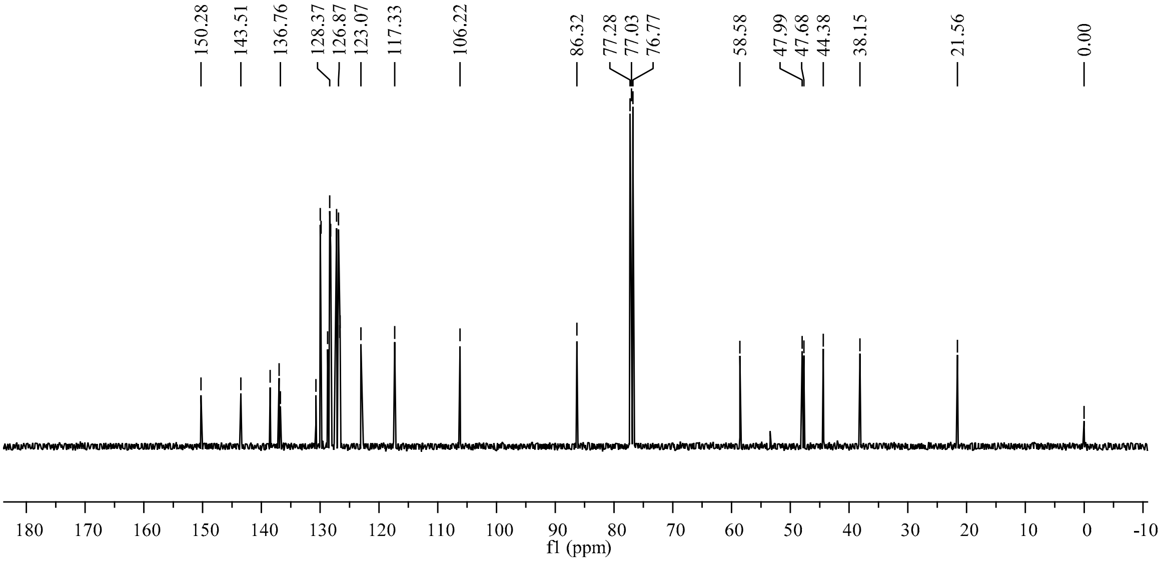

Figure S12. ^{13}C-NMR spectroscopic data for compound **a21**.

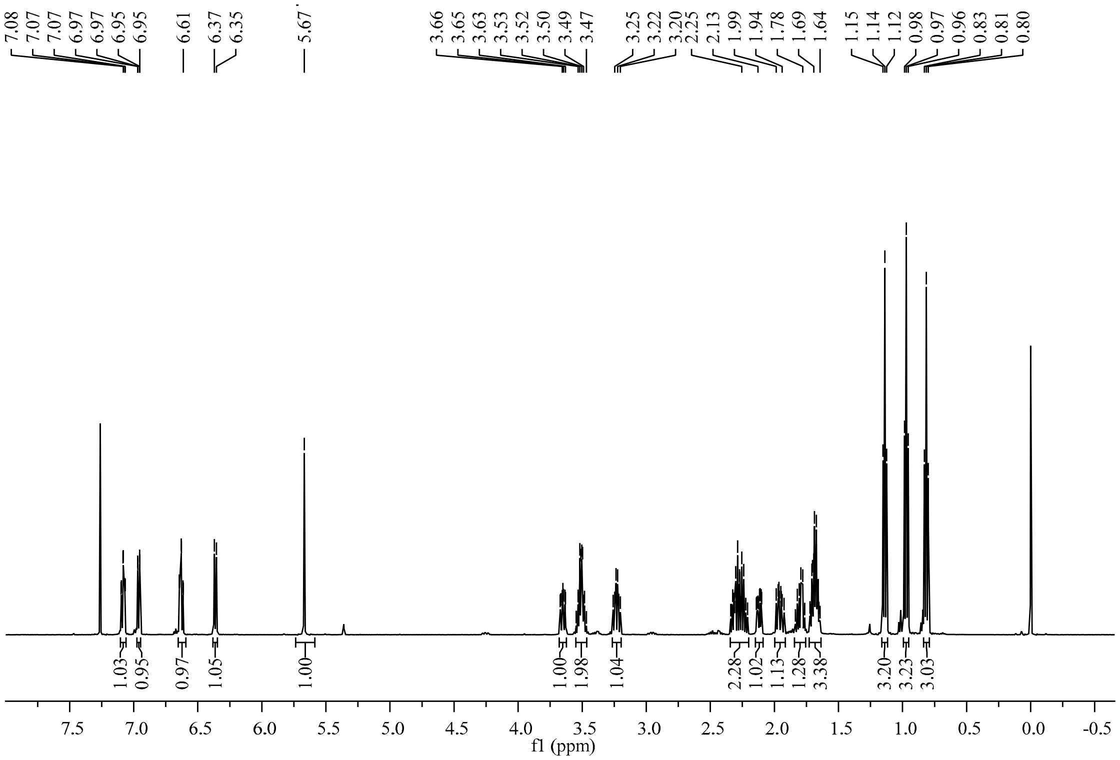

Figure S13. ^{1}H-NMR spectroscopic data for compound **b3**.

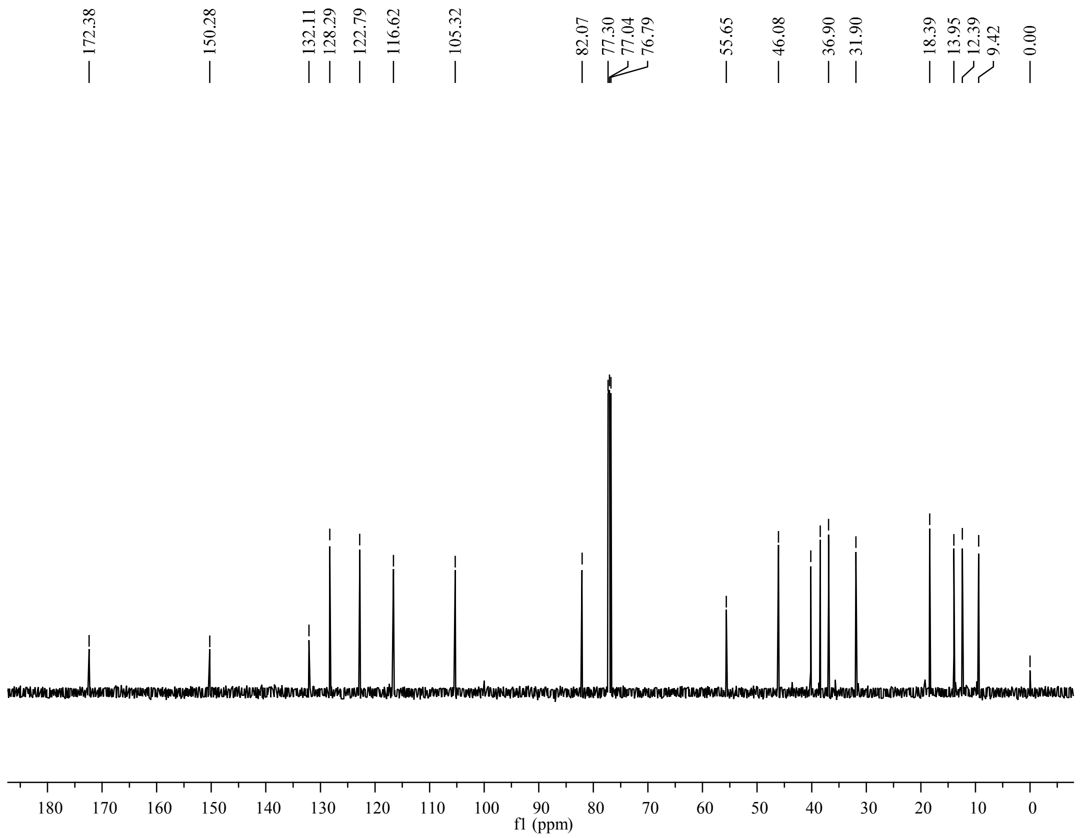

Figure S14. ^{13}C-NMR spectroscopic data for compound **b3**.

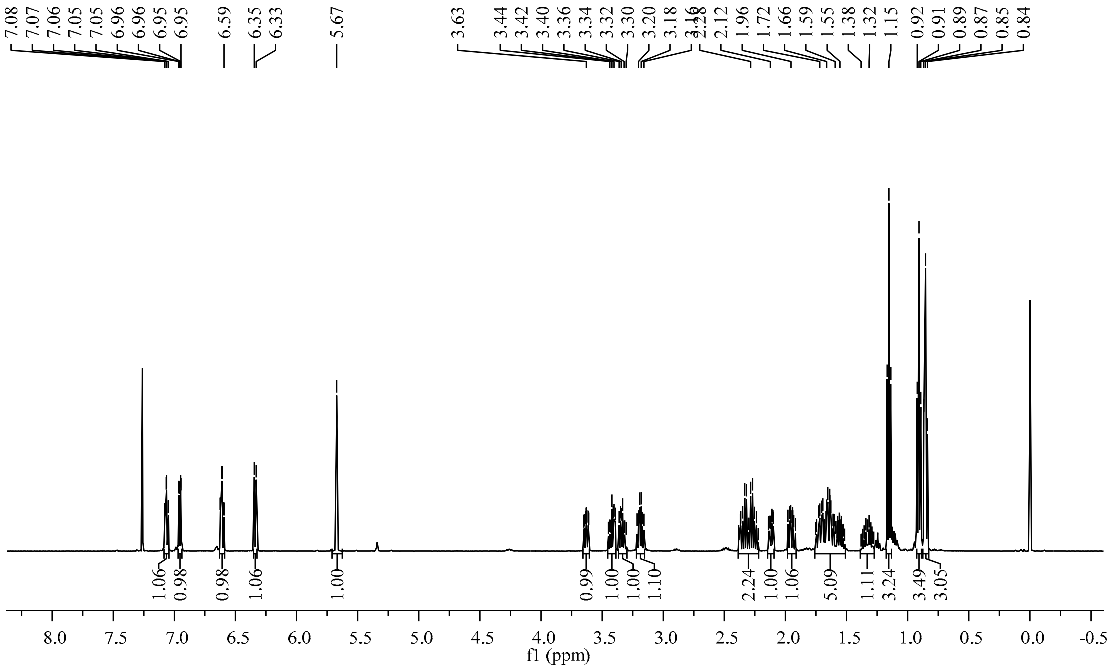

Figure S15. ^{1}H-NMR spectroscopic data for compound **c2**.

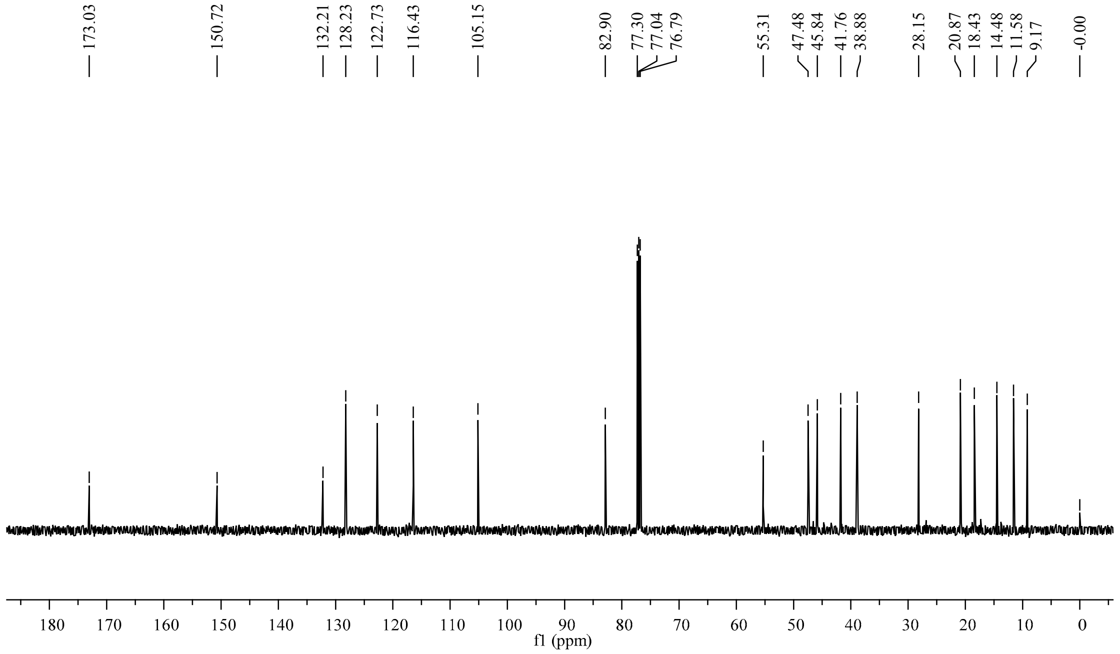

Figure S16. ^{13}C-NMR spectroscopic data for compound **c2**.
